# Nuclear activities and interactome of the NS5 protein of tick-borne encephalitis virus

**DOI:** 10.1128/jvi.00810-26

**Published:** 2026-08-10

**Authors:** Maxime Chazal, Aïssatou Aïcha Sow, Elodie Le Seac'h, Dana Hawasheen, Margarida Bonifacio, Mikael Feracci, Ségolène Gracias, Adrià Sogues, Melissa Molho, Heng Pan, Boris Bonaventure, Jean-Pierre Quivy, Geneviève Almouzni, Etienne Decroly, Holly Ramage, Jeffrey R. Johnson, Vincent Caval, Nolwenn Jouvenet

**Affiliations:** 1 Unité Signalisation Antivirale, UMR CNRS 3569, Université Paris Cité, Institut Pasteur, Paris, France; 2 Aix Marseille Université, AFMB, UMR CNRS 7257 56164 , Marseille, France; 3 Bacterial Cell Cycle Mechanisms Unit, UMR CNRS 3528, Université Paris Cité, Institut Pasteur 555089, Paris, France; 4 Department of Microbiology and Immunology, Thomas Jefferson University 6559, Philadelphia, Pennsylvania, USA; 5 Department of Microbiology, Icahn School of Medicine at Mount Sinai 5925, New York, New York, USA; 6 Global Health and Emerging Pathogens Institute, Icahn School of Medicine at Mount Sinai 5925, New York, New York, USA; 7 Institut Curie, PSL Research University, Sorbonne Université, UMR CNRS 3664, Laboratoire Dynamique du Noyau, Equipe Labellisée Ligue contre le Cancer 55216, Paris, France; University of North Carolina at Chapel Hill, Chapel Hill, North Carolina, USA

**Keywords:** tick-borne encephalitis virus, NS5 interactomics, NS5 protein, orthoflaviviruses

## Abstract

Orthoflaviviruses are RNA viruses responsible for significant diseases in humans, domesticated animals, and wildlife. Their NS5 protein is central in viral replication, functioning both as an RNA-dependent RNA polymerase and a methyltransferase, while also modulating cellular processes, including the interferon response. Although viral replication is cytoplasmic, the NS5 protein of several mosquito-borne orthoflaviviruses cycles between the cytoplasm and the nucleus of infected human cells. However, the nuclear localization and function of NS5 of tick-borne orthoflaviviruses, such as tick-borne encephalitis virus (TBEV), remain poorly understood. Microscopy analysis and cell fractionation revealed that the NS5 protein of TBEV localized to both the cytoplasm and nucleoplasm of infected cells. Mutagenesis studies identified critical residues required for its nuclear targeting. Mutating these residues in a TBEV replicon abolished viral replication. Immunoprecipitation-mass spectrometry analyses performed in two human cell lines infected with TBEV recovered 352 NS5 partners. Among them, 187 were nuclear or partially nuclear. By integrating our interactome data with that of Powassan virus (POWV), another tick-borne orthoflavivirus, we refined a list of 20 high-confidence NS5 partners, including splicing factors and chromatin modulators. Functional analysis revealed that seven of these nuclear partners significantly modulated viral replication, further underscoring the importance of nuclear NS5 in the viral life cycle. Our work advances our understanding of the nuclear function of the NS5 proteins of tick-borne orthoflaviviruses.

**IMPORTANCE:**

Tick-borne orthoflaviviruses are emerging globally, spreading across Europe, Asia, and North America, where they infect humans, domesticated animals, and wildlife. These viruses produce a protein called NS5, which drives viral replication and helps evade the innate immune response. We observed that the NS5 protein of tick-borne encephalitis virus (TBEV) localized both in the cytoplasm and nucleoplasm of infected human cells. We identified the specific residues responsible for its nuclear addressing and showed that it interacts with numerous nuclear proteins, including some involved in regulating gene expression. Seven of these nuclear partners significantly influenced viral replication, highlighting the importance of NS5’s nuclear activity. This work sheds light on how tick-borne orthoflaviviruses manipulate host cells, deepening our understanding of their replication strategies.

## INTRODUCTION

Most orthoflaviviruses are primarily transmitted to vertebrate hosts by mosquitoes or ticks (1). They have a worldwide distribution and infect up to 400 million people annually. They cause a wide spectrum of diseases, including hepatitis, vascular leakage, shock syndrome, encephalitis, acute flaccid paralysis, congenital abnormalities, and death (1). Over the past 50 years, outbreaks of orthoflaviviruses have increased significantly. For instance, the introduction into the Western Hemisphere of West Nile virus (WNV) in 1999 and Zika virus (ZIKV) in 2015 has led to large numbers of severe human infections (2, 3). Dengue fever, which is caused by one of the four serotypes of dengue virus (DENV), is currently experiencing an unprecedented worldwide resurgence (4). Meanwhile, Yellow fever virus (YFV) outbreaks, driven by insufficient vaccination coverage, remain a major concern (5). Finally, tick-borne orthoflaviviruses are also emerging in various parts of the world, as seen with tick-borne encephalitis virus (TBEV) in Europe and Powassan virus (POWV) in North America (6).

Orthoflaviviruses share a common genome organization and coding strategy (1). Upon viral entry into the host cell and fusion between the viral and endosomal membranes, the 11-kb viral genome is released into the cytosol and addressed to the endoplasmic reticulum (ER) to be translated by the cellular machinery into a large polyprotein precursor. This polyprotein is processed by host and viral proteases into three structural proteins (the capsid protein [C], the precursor of the M protein [prM], and the envelope [E] glycoprotein) and seven non-structural (NS) proteins (NS1, NS2A, NS2B, NS3, NS4A, NS4B, and NS5) (1). The structural proteins form the virus particles, whereas the NS proteins are involved in viral replication and assembly, as well as in counteracting the cellular antiviral response (1).

The orthoflavivirus NS5 protein is a highly conserved protein of about 100 kDa. It contains two enzymatic domains, an N-terminal methyltransferase (MTase) domain and an RNA-dependent RNA polymerase (RdRp) domain, which are responsible for capping and replication of the viral genome, respectively (7). NS5 is also a potent inhibitor of interferon (IFN) signaling (8, 9). Although viral replication occurs within the cytoplasm, the NS5 protein of several mosquito-borne orthoflaviviruses, including DENV and ZIKV, localizes to the nucleus of infected human cells (10–12). DENV NS5 carries two highly conserved nuclear localization signals (NLS) that are recognized by the importin β1 and importin α/β nuclear import factors (10, 13), with the αβNLS likely being the primary determinant of nuclear addressing (10). ZIKV NS5, which localizes mainly in punctate nuclear bodies in infected cells, also interacts with importin β1 and importin α/β (11, 12).

Several functions have been attributed to the nuclear localization of the NS5 protein of mosquito-borne orthoflaviviruses. Nuclear translocation may protect NS5 from proteolytic degradation in the cytoplasm (11). Nuclear NS5 may also exert proviral functions via interactions with nuclear partners. Indeed, proteomic analysis coupled to functional studies in human hepatocellular carcinoma Huh7 cells and embryonic kidney 293T cells infected with DENV2 revealed that NS5 perturbs the correct splicing of antiviral factors by interacting with core components of the splicing machinery (14). Similarly, proteomic analysis using 293T cells expressing tagged versions of the NS5 protein of ZIKV, DENV2, WNV, and Japanese encephalitis virus (JEV) recovered numerous spliceosomal components and spliceosome-associated proteins (15–17), suggesting that NS5-mediated targeting of the spliceosome is a strategy shared by mosquito-borne orthoflaviviruses to modulate gene expression. Finally, ZIKV NS5 blocks the transcriptional elongation of numerous genes via direct binding to chromatin, leading to a disruption of neurogenesis in the mouse brain (18). In tick-borne orthoflaviviruses, the nuclear localization of NS5 and its functional consequences remain unclear.

We recently produced an antibody against TBEV NS5 and observed, using confocal microscopy analysis, that the protein localized both in the cytoplasm and nucleoplasm of infected Huh7 cells (9). We aimed here to better understand what controls the nuclear presence of TBEV NS5 and get insight into its functional role. Using mutagenesis approaches and immunoprecipitation-mass spectrometry (IP-MS) of infected cells coupled to functional studies, we identified residues critical for NS5 nuclear localization and provided a list of NS5 nuclear partners, some of which modulate viral replication.

## RESULTS

### The nuclear accumulation of TBEV NS5 requires importin β1- and α/β-interacting domains

**Fig 1:**
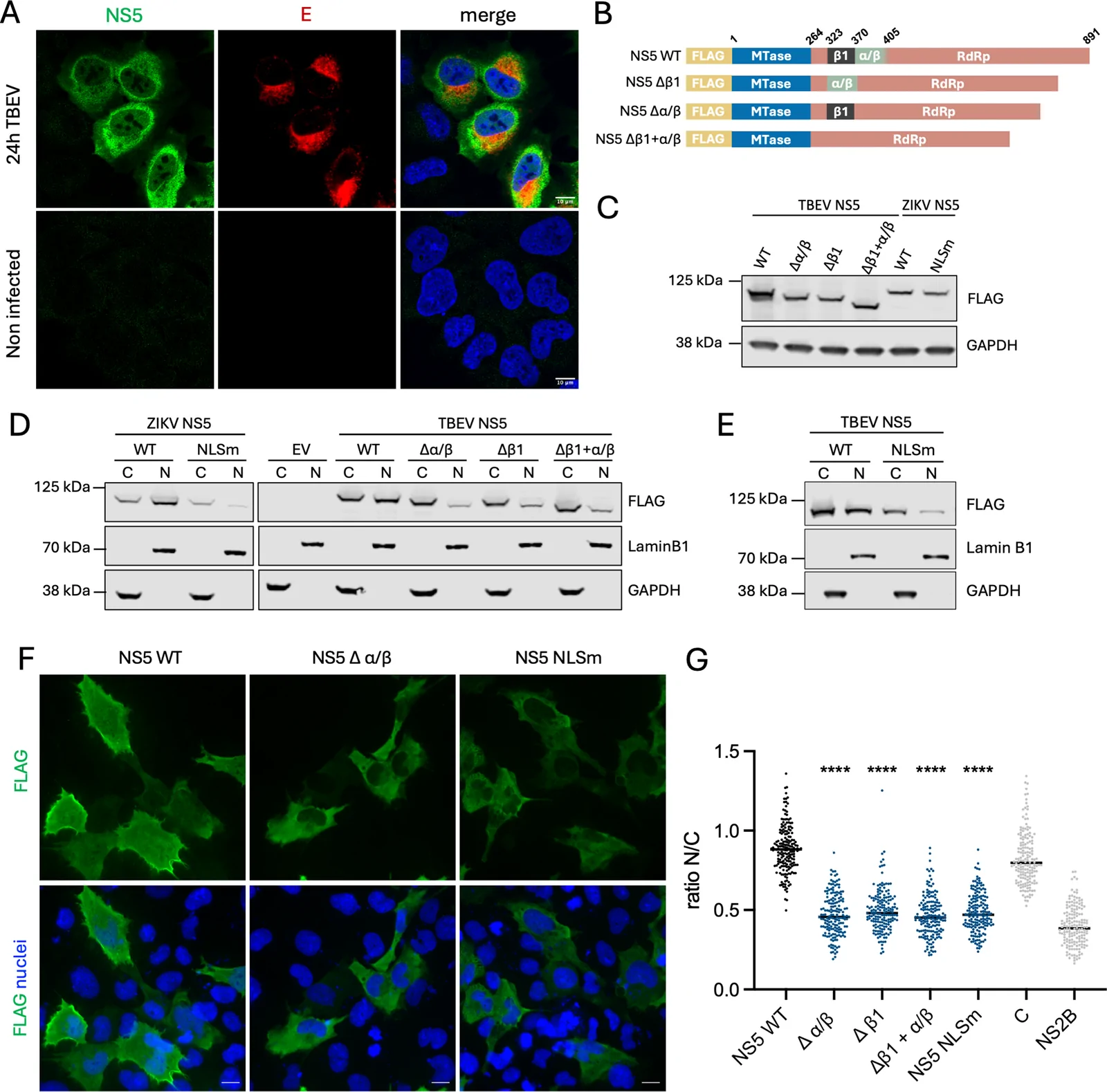
Characterization of TBEV NS5 nuclear localization. (**A**) HMC3 cells were mock-infected or infected with TBEV at an MOI of 1. After 24 h, subcellular localization of NS5 was determined by immunofluorescence microscopy using antibodies against NS5 (green) or E (red). Nuclei were stained using DAPI (blue), and images were acquired using a confocal microscope. Scale bars = 10 µm. (**B**) Schematic representation of wild-type (WT) and mutant NS5 proteins. (**C**) 293T cells were transfected with plasmids encoding TBEV or ZIKV FLAG-NS5 WT or mutants. After 24 h, whole-cell lysates were analyzed by Western blot using antibodies against FLAG and GAPDH. (**D and E**) 293T cells were transfected with plasmids encoding TBEV or ZIKV FLAG-NS5, WT, or mutants. After 24 h, NS5 subcellular localization was determined by cellular fractionation. Cytoplasmic (C) and nuclear (N) fractions were analyzed by Western blot using antibodies against FLAG, GAPDH, and Lamin B1. (**F**) HMC3 cells were transfected with plasmids expressing TBEV FLAG-tagged NS5 WT, Δα/β, or NLSm. After 24 h, subcellular localization of NS5 was determined by immunofluorescence microscopy using an antibody recognizing FLAG (green). Nuclei were stained using DAPI (blue), and images were acquired using an epifluorescence microscope. Scale bars = 10 µm. (**G**) Quantification of the nuclear localization of NS5 WT or mutants was determined by measuring the intensity of cytoplasmic (C) and nuclear (N) FLAG signal from images shown in panel F and Fig. S2 using Fiji. FLAG-tagged TBEV C and NS2B proteins were included as controls. The ratio of mean fluorescence N/C is represented. Data are from 164 to 193 cells across three independent experiments for each condition. Statistical analysis was performed using an unpaired *t*-test to compare NS5 WT to NS5 mutants, ****P < 0.0001.

We previously observed, using an in-house generated TBEV NS5 antibody, that TBEV NS5 localized both in the cytoplasm and nucleus of Huh7 cells infected for 48 h with TBEV (Hypr strain) at an MOI of 0.02 (9). We confirmed the localization of NS5 using human microglial cells (HMC3), which are relevant for TBEV biology since they are sites of viral replication *in vivo* (19). As in Huh7 cells (9), TBEV NS5 exhibited a diffuse cytoplasmic and nuclear localization in HMC3 cells infected at an MOI of 1 for 24 h (Fig. 1A). The NS5 signal was excluded from the nucleolus in infected HCM3 (Fig. 1A) and Huh7 cells (Fig. S1A). By contrast, the E signal was cytoplasmic (Fig. 1A).

The NLS of the NS5 proteins of several mosquito-borne orthoflaviviruses have been characterized. Both DENV and ZIKV NS5 carry a bipartite NLS that interacts with importin α/β and importin β1, with the importin α/βNLS being the predominant one for DENV2 NS5 (10–13). Using alignment of the NS5 proteins of TBEV, POWV, ZIKV, DENV2, JEV, and WNV (Fig. S1B), we found that TBEV NS5 also contains basic residues within the N-terminal region of its RdRp domain that may bind importin β1 and importin α/β and thus could serve as an NLS. To determine the impact of these two importin-binding domains on TBEV NS5 nuclear accumulation, we generated versions of the protein lacking importin β1-binding residues (Δβ1), importin α/β-binding residues (Δα/β), or both domains (Δβ1+α/β). Wild-type TBEV NS5 and the three deletion mutants were FLAG-tagged at their N-terminus (Fig. 1B). A FLAG-tagged version of wild-type ZIKV NS5 (strain H/PF/2013), which localizes in the nucleus of infected Huh7 cells (12), was included in the analysis as a comparison. We also generated, based on previous data obtained with the ZIKV strain MR766 (12), a cytoplasmic mutant of ZIKV NS5 H/PF/2013 (referred to herein as NS5-_ZIKV_NLSm), in which two positively charged residues (K390 and R393) of the NLS were mutated to alanine. 293T cells, which are easy to transfect, were used to assess the intracellular distribution of wild-type and mutant NS5 proteins of TBEV and ZIKV. Western blot analysis revealed that all the expressed NS5 TBEV mutants migrated at their expected molecular weight but were produced at slightly lower levels than their wild-type counterparts (Fig. 1C). Similarly, reduced expression of the NS5-_ZIKV_NLSm, as compared to ZIKV NS5 wild type, was previously observed in transfected 293T cells (11). These data, together with ours (Fig. 1C), suggest that the importin-binding sites could be involved in NS5 stability.

In line with previous microscopic analysis performed in 293T cells (12), cell fractionation experiments revealed that the majority of ZIKV NS5 wild type was found in the nuclear fraction when expressed on its own. In contrast, the NS5-_ZIKV_NLSm was barely detected in the nuclear fraction (Fig. 1D). As in HMC3 cells infected with TBEV for 24 h (Fig. 1A), similar amounts of exogenously expressed wild-type TBEV NS5 protein were found in the cytoplasmic and nuclear fractions of 293T cells (Fig. 1D). Deletion of β1NLS, α/βNLS, or both domains shows a similar amount of NS5 in the cytoplasm, but reduced the amount of NS5 in the nucleus (Fig. 1D), suggesting that these two domains are involved in TBEV NS5 nuclear import. To identify the critical residues that are required for TBEV NS5 nuclear localization, all the basic residues within the two importin-binding domains were mutated to alanine. They were mutated either individually, in pairs, or in threes when near each other (Fig. S1C). Expression levels of the mutants were similar, suggesting that the point mutations had little impact on protein stability, with the exception of the double mutant R327A + R376A and the triple mutant K389A + K391A + R393A, which were expressed at approximately twofold and threefold lower levels than the other mutants, respectively (Fig. S1D). All single and double mutants, like wild-type NS5, were equally distributed between the cytoplasmic and nuclear fractions (Fig. S1E). By contrast, the triple mutant K389A + K391A + R393A, which we named the NS5-_TBEV_NLSm mutant, was predominantly in the cytoplasmic fraction (Fig. 1E).

The cellular distribution of FLAG-tagged versions of NS5 WT, as well as the four mutants (Δα/β, Δβ1, Δβ1+ α/β, and NS5-_TBEV_NLSm), was assessed by epifluorescence microscopy in HMC3 cells (Fig. 1F; Fig. S2). FLAG-tagged versions of NS2B and C proteins, which are known to localize to the cytoplasm and to both the nucleus and the cytoplasm, respectively (20, 21), were included in the analysis as controls (Fig. S2). The nuclear-to-cytoplasmic (N/C) ratio for NS5 was approximately 0.9 (Fig. 1G). This ratio was similar for the C protein (Fig. 1G). In contrast, the N/C ratio was around 0.4 for the four NS5 mutants, similar to that of NS2B. These findings confirmed that the four NS5 mutants exhibited a significantly reduced nuclear localization as compared to NS5 WT (Fig. 1G; Fig. S1E).

**Fig 2:**
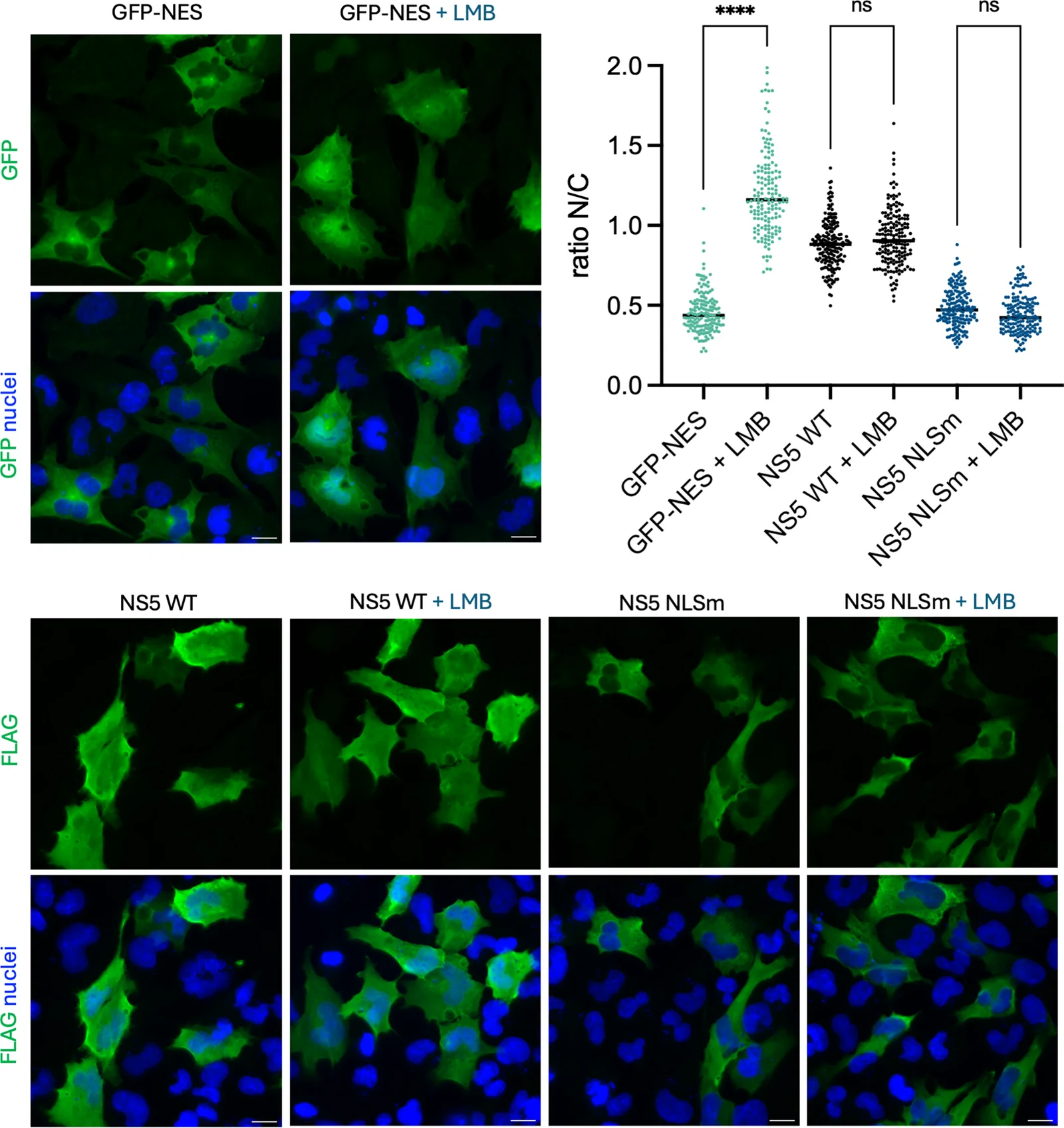
CRM1 inhibition fails to induce nuclear accumulation of TBEV NS5. HMC3 cells were transfected with plasmids expressing GFP-NES, FLAG-tagged NS5 WT, or NS5-_TBEV_NLSm. After 24 h, cells were treated for 2 h with leptomycin B (LMB, 10 nM), and subcellular localization of NS5 was determined by immunofluorescence microscopy using an antibody recognizing FLAG (green). Nuclei were stained using DAPI (blue), and images were acquired using a brightfield microscope. Scale bars = 10 µm. Quantification of nuclear localization was determined by measuring the intensity of the cytoplasmic (C) and nuclear (N) signals using Fiji. The ratio of mean fluorescence N/C is represented. Data are from 162 to 193 cells across three independent experiments for each condition. Statistical analysis was performed using an unpaired *t*-test to compare mock-treated cells to LMB-treated cells, ****P < 0.0001. Scale bars = 10 µm.

To investigate further NS5 nuclear trafficking, HMC3 cells expressing FLAG-tagged NS5 WT or NS5-_TBEV_NLSm were treated for 2 h with leptomycin B (LMB), an inhibitor of CRM1-mediated nuclear export (Fig. 2). Cells expressing GFP fused to a nuclear export signal (NES) known to bind CRM1 served as positive controls (22). As expected (22), GFP-NES accumulated in the nucleus in the presence of LMB (Fig. 2). In contrast, the distribution of NS5 WT and NS5-_TBEV_NLSm was unaffected by LMB (Fig. 2), suggesting that TBEV NS5 is exported from the nucleus in a CRM1-independent manner, as described for other viral proteins such as adenovirus E4orf6 (23).

Together, our data identified three residues (K389, K391, and R393) that are essential for the nuclear import of TBEV NS5 and suggest that its nuclear export is independent of CRM1.

### Disruption of the NS5 NLS impairs protein stability and viral replication

**Fig 3:**
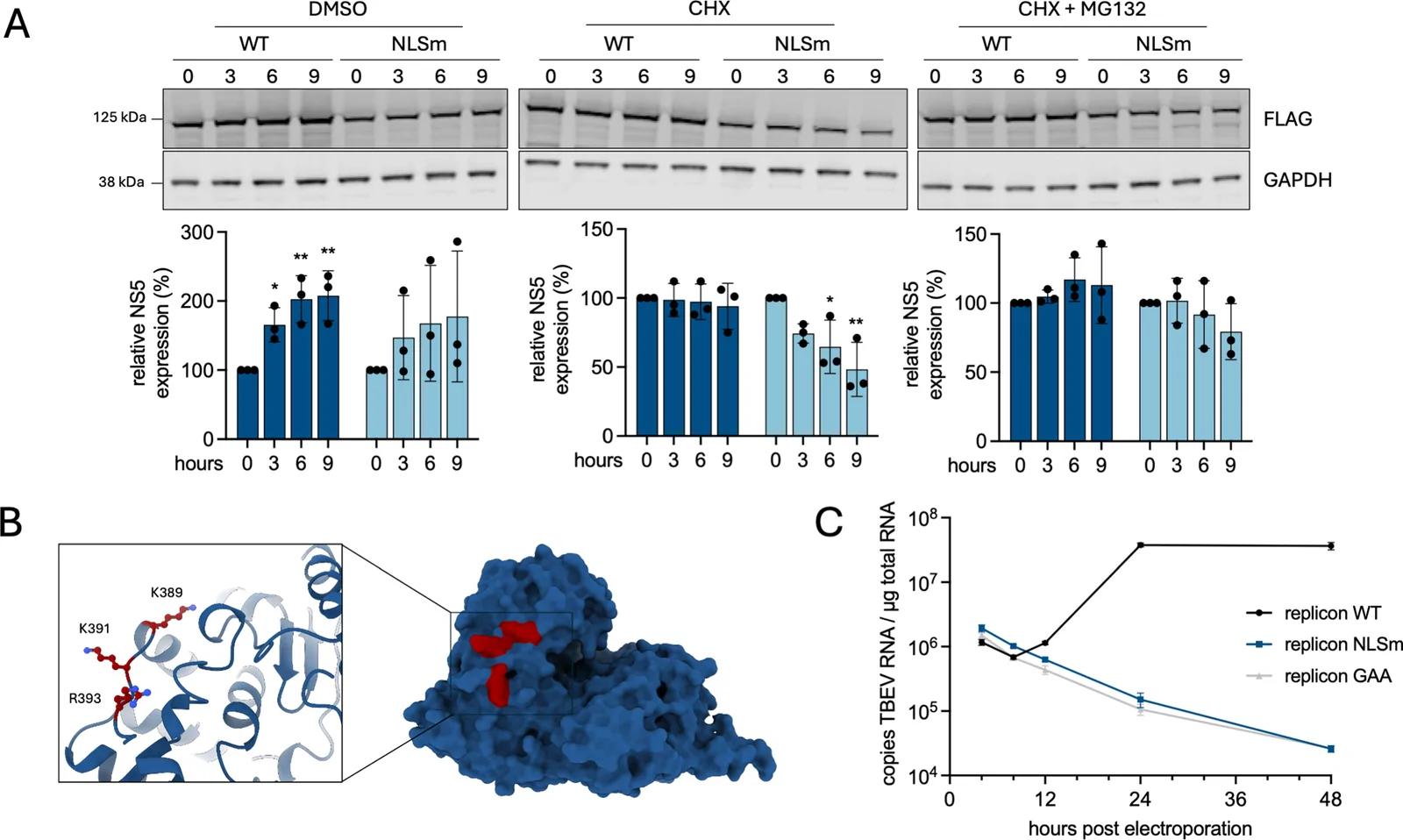
Disruption of the NS5 NLS impairs protein stability and viral replication. (**A**) 293T cells expressing TBEV FLAG-tagged NS5 (WT or NLSm) were treated with CHX alone or in combination with MG132 for 3, 6, or 9 h. DMSO-treated cells were used as a control. Bottom panels show the quantitative analyses of the effects of the treatments. Unpaired *t*-test was performed to compare mock-treated cells to CHX- or CHX + MG132-treated cells, *P < 0.05, **P < 0.01. (**B**) AlphaFold model of the RdRp domain of TBEV NS5. The three amino acids essential for nuclear import are highlighted in red. (**C**) The effect of NS5 nuclear localization on viral replication was assessed by mutating the NLS signal in a TBEV replicon. *In vitro* transcribed RNAs from WT or mutated replicons were electroporated into HMC3 cells, and viral replication was measured using RT-qPCR.

Our results suggest that NS5-_TBEV_NLSm is less stable than the wild-type NS5 (Fig. S1D). To study further the stability of the two proteins, we compared their degradation kinetics in the presence of cycloheximide (CHX) alone or in combination with MG132. CHX is an inhibitor of protein translation, and MG132 is an inhibitor of proteasome-mediated degradation. 293T cells expressing NS5 WT or NS5-_TBEV_NLSm were treated with CHX and/or MG132 for 3, 6, or 9 h, or with DMSO as a control for the same durations (Fig. 3A). In DMSO-treated cells, NS5 levels increased significantly over time, whereas NS5-_TBEV_NLSm levels exhibited a modest non-significant increase (Fig. 3A). In CHX-treated cells, NS5 levels remained constant, while NS5-_TBEV_NLSm levels significantly decreased over time. When cells were treated with both CHX and MG132, the levels of both proteins remained relatively stable (Fig. 3A). These results further suggest that NS5-_TBEV_NLSm is less stable than the wild-type protein and indicate that it may be degraded via the proteasome.

To explore the importance of the NS5 NLS for TBEV replication, we took advantage of a replicon derived from the Neudoerfl strain (24), in which we mutated the three critical positively charged residues of the α/βNLS to alanine (replicon NLSm). These three residues are predicted to be surface-exposed (Fig. 3B). We included in the analysis a wild-type replicon and a replication‐defective replicon bearing double D‐to‐A mutations in the RdRp catalytic sequence GDD (replicon‐GAA) (9), as positive and negative controls, respectively. *In vitro* transcripts synthesized from plasmids coding for these three replicons were electroporated into HMC3 cells, and viral RNA levels were measured by RT-qPCR analysis at various time points post-electroporation. As expected, the wild-type replicon produced increasing amounts of viral RNA over time, whereas the replicon‐GAA did not (Fig. 3C). RNAs derived from the NSLm replicon were unable to replicate (Fig. 3C), indicating that the three NLS residues, and potentially nuclear localization of NS5, are critical for viral replication.

### The TBEV NS5 interactome is enriched in nuclear proteins

**Fig 4:**
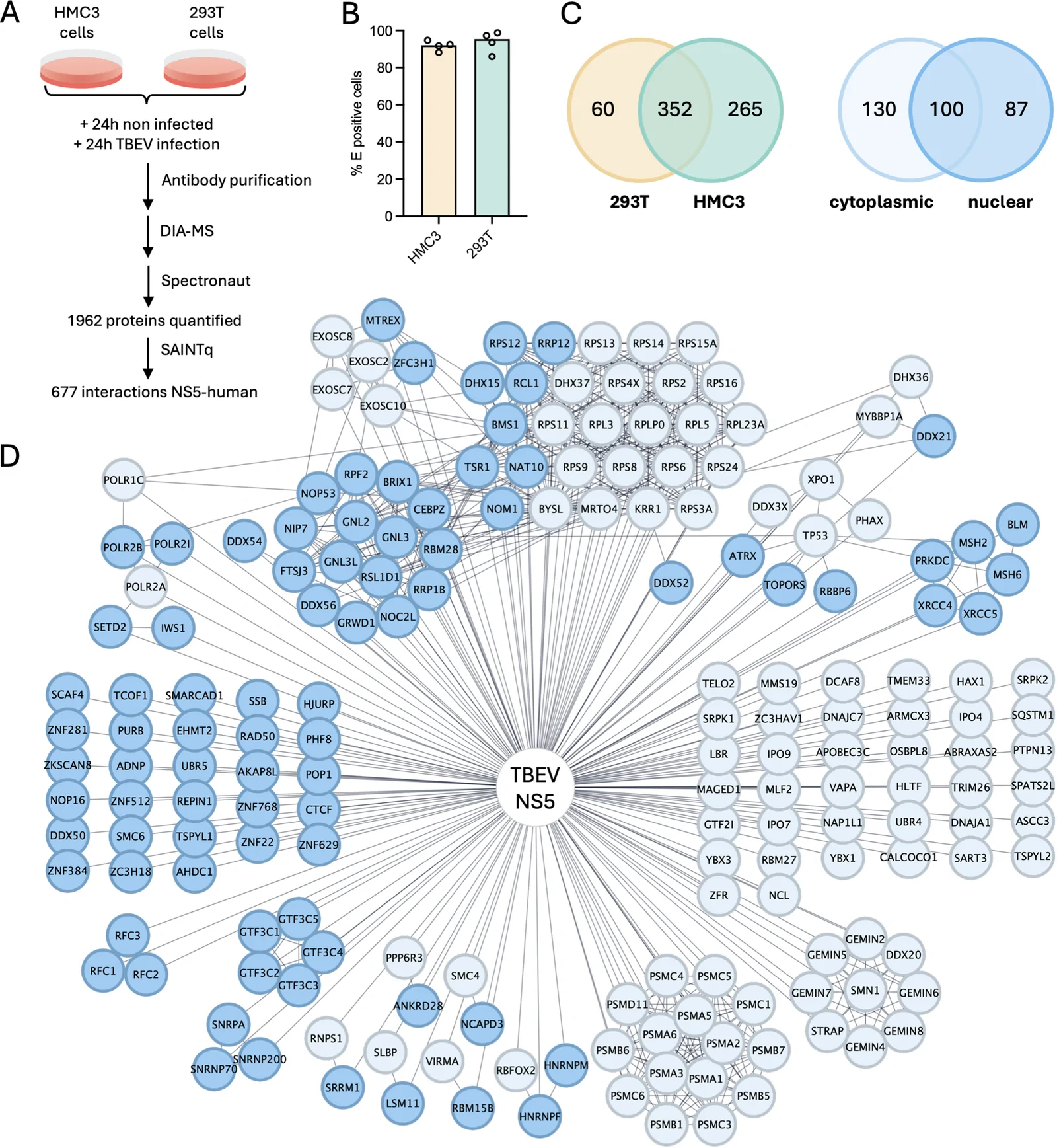
Identification of NS5 partners during TBEV infection. (**A**) Experimental flow. HMC3 and 293T cells were mock-infected or infected with TBEV. After 24 h, cell lysates were immunoprecipitated with anti-NS5 coupled to magnetic beads. Immunoprecipitated samples were analyzed by mass spectrometry using four independent biological replicates. Proteomic data were analyzed using SAINTq for interaction scoring. (**B**) The percentage of infected cells at 24 h post-infection was assessed by flow cytometry analysis using antibodies against the viral E protein. (**C**) Venn diagrams of TBEV NS5-interacting partners identified by MS. The left panel shows the overlap of NS5 partners between the two cell lines. The right panel depicts the subcellular localization of 327 partners (out of the 352 identified in both cell lines), as determined by UniProt protein ID annotations. Of note, 25 proteins had unknown subcellular localization. (**D**) Nuclear partners of TBEV NS5 identified in both HMC3 and 293T cells are represented. Partners with exclusive nuclear localization are depicted in dark blue, while those with partial nuclear localization are depicted in light blue. Protein–protein interaction networks were mapped using STRING analysis.

To identify cellular partners of TBEV NS5 during infection, we performed IP-MS in 293T and HMC3 cells infected for 24 h with the Hypr strain at an MOI of 1 (Fig. 4A). In these conditions, at 24 h post-infection (hpi), approximately 90% of cells were positive for the viral protein E (Fig. 4B). TBEV NS5 was purified from infected cells using an in-house NS5 antibody. Non-infected cells were used as negative controls. The eluates were first analyzed by silver staining (Fig. S3A) and then by data-independent acquisition mass spectrometry (DIA-MS). Over 1,900 host proteins were detected in the purified samples from both cell lines (Fig. S3B). NS5 protein intensities in infected cells exceeded over 256-fold those of mock-infected controls (Fig. S3C). Pairwise correlation analysis confirmed the reproducibility of protein intensity profiles across biological replicates (Fig. S3D). We further analyzed the data with SAINTq, an interaction scoring algorithm optimized for DIA-MS data (25). A total of 677 proteins, which had a Bayesian False Discovery Rate (BFDR) < 0.05, were considered potential partners of TBEV NS5 (Table S1). Global proteomics analyses performed on mock- versus TBEV-infected cells (Table S2) confirmed that host proteins identified as NS5 partners were not upregulated, excluding their enrichment in IP due to overexpression.

Among these partners, 352 proteins were common to both cell lines (Fig. 4C). Sixteen of these were recently identified as NS5 TBEV partners in an MS analysis performed in transfected 293T cells (26), such as SETD2 and NKTR. Protein ID inquiries using UniProt revealed that most of these 352 partners localize exclusively or partially to the nucleus (Fig. 4C), consistent with the nuclear localization of NS5 (Fig. 1). Gene Ontology analysis of the TBEV NS5 nuclear partners showed that they participate in chromosome organization and remodeling, as well as RNA splicing, methylation, and metabolism, such as the members of the Gemin-SNM complex (27) (Fig. 4D; Fig. S4). This suggests that TBEV NS5 may modulate host epigenetic, transcriptomic, and epitranscriptomic programs to facilitate viral replication. Additionally, numerous ribosomal proteins, as well as about 15 proteasomal proteins, were identified, suggesting that NS5 may also associate with these complexes (Fig. 4D). Several NS5 interactors, such as ATRX and ZRANB2, were previously identified by MS analyses as partners of ZIKV, DENV, and WNV NS5 (16, 17), indicating that these proteins are conserved partners of orthoflavivirus NS5.

**Fig 5:**
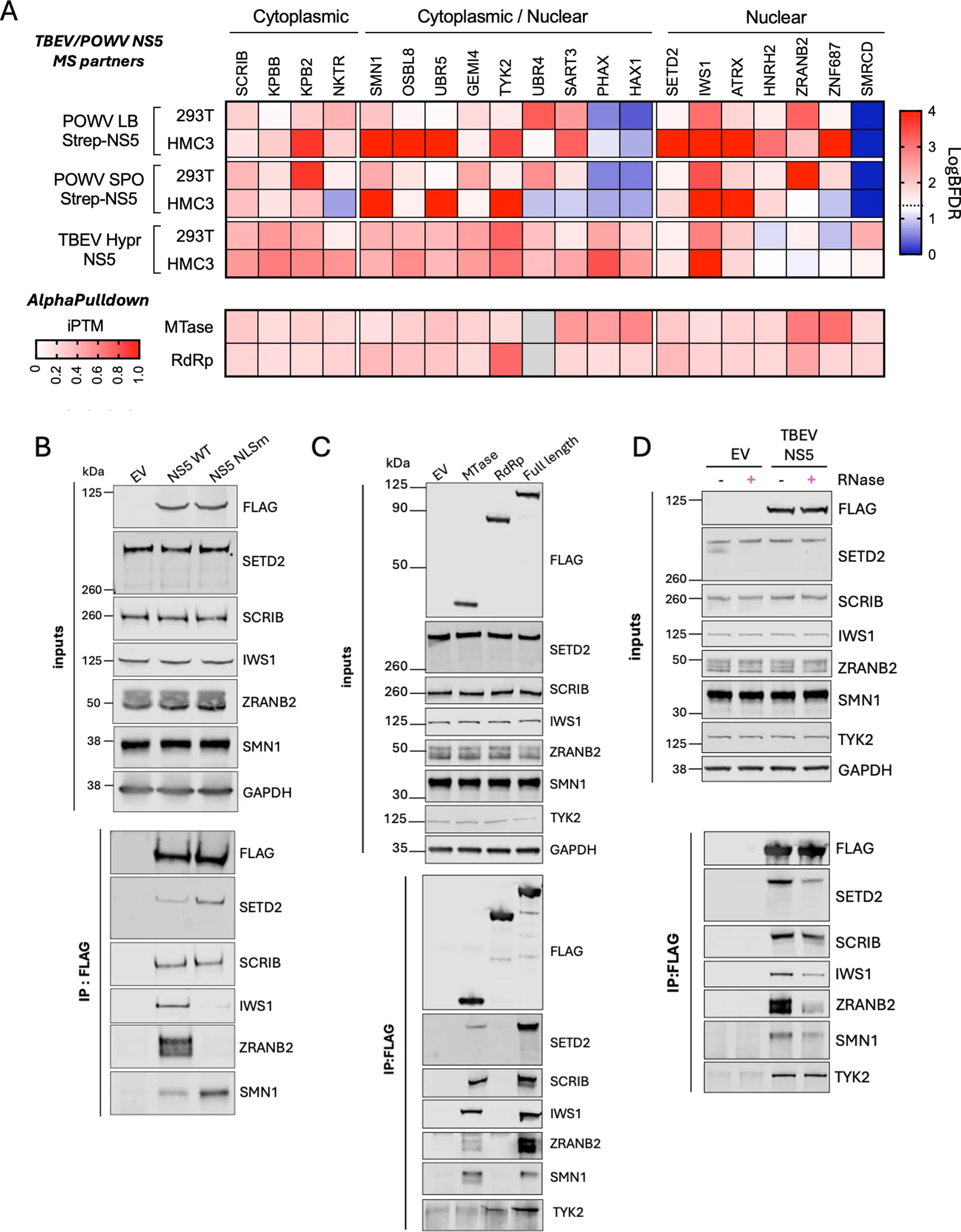
Validation of NS5 interaction with high-confidence nuclear and cytoplasmic partners. (**A**) Upper panel: heat map representation of LogBFDR scores of NS5-interacting proteins identified by MS for TBEV and POWV NS5. Proteins with LogBFDR > 1.3 in at least four of the six data sets are displayed and considered as high-confidence NS5 partners. Lower panel: heat map of iPTM scores from an AlphaPulldown screen between TBEV-NS5 MTase or RdRp domains and the top 20 host protein partners. The iPTM value for UBR4 was not computed, as the protein size exceeded the computationally allowed limit. (**B**) 293T cells were transfected for 24 h with plasmids encoding FLAG-tagged WT or NLSm NS5 TBEV proteins, or an empty vector (EV) as a control. To ensure comparable expression levels between the two viral proteins, cells received four times the amount of plasmids coding for NS5-NLSm compared to the ones transfected with wild-type NS5 plasmids. Cell lysates were immunoprecipitated using FLAG antibodies. Western blot analysis was performed on whole-cell lysates (inputs) and FLAG-IP using the indicated antibodies. (**C**) 293T cells were transfected for 24 h with plasmids encoding FLAG-tagged MTase, RdRp, or full-length NS5 TBEV proteins, or an empty vector as a control. Cell lysates were immunoprecipitated using FLAG antibodies. Western blot analysis was performed on whole-cell lysates (inputs) and FLAG-IP using the indicated antibodies. (**D**) 293T cells were transfected for 24 h with plasmids encoding FLAG-tagged NS5 TBEV protein, or an empty vector as a control. Cell lysates were treated with RNase A or left untreated and were immunoprecipitated using FLAG antibodies. Western blot analyses were performed on whole-cell lysates (inputs) and FLAG-IP using the indicated antibodies.

To assess the conservation of these interactions across tick-borne orthoflaviviruses, we compared our TBEV NS5 interactome with that of POWV NS5, which was identified through affinity purification (AP) MS in 293T and HMC3 cells expressing Strep-tagged NS5 proteins of 2 POWV strains (LB and SPO) (M. Molho, H. Pan, B. Bonaventure, H. Ramage, and J. R. Johnson, unpublished data). Seventeen proteins were shared across at least four of the six data sets (Fig. 5A, top panel). Since these candidates were identified in two cell lines (293T cells and HMC3), either during infection with TBEV or in cells expressing POWV NS5, they were considered high-confidence partners of tick-borne orthoflavivirus NS5. Among these, SCRIB and TYK2 were previously identified by us and others as direct partners of TBEV NS5 in a variety of cellular models, including yeasts and human cell lines (9, 28, 29), further validating our approach. We were also interested in potential TBEV NS5-specific partners. Three nuclear proteins (PHAX, HAX1, and SMRCD), which emerged as top candidates based on their BFDR scores in 293T and HMC3 cells infected with TBEV (Fig. 5A, top panel), were not identified as POWV NS5 partners. These three candidates, along with the seventeen shared proteins (excluding TYK2, whose role in viral replication is already well characterized [9]), were selected for further functional investigations.

**Fig 6:**
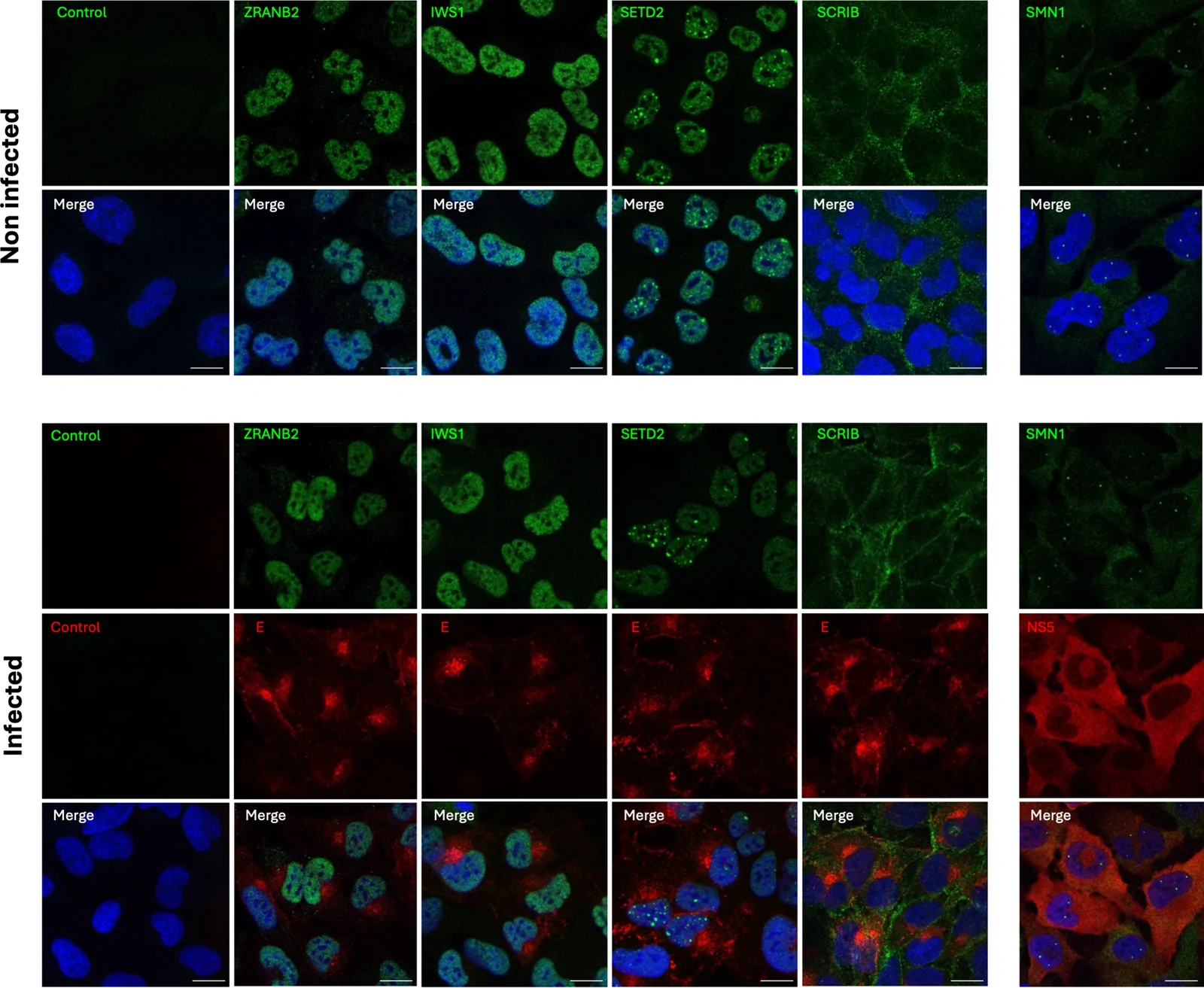
Impact of TBEV infection on the subcellular localization of a subset of NS5 partners. HMC3 cells were infected or not infected with TBEV at an MOI of 1. After 24 h, the localization of selected TBEV NS5 partners was analyzed by immunofluorescence microscopy using specific antibodies (green). Infected cells were marked with antibodies against E or NS5 (red), based on species compatibilities. Nuclei were stained using DAPI (blue), and images were acquired using a confocal microscope. Acquisition settings were based on cells stained with secondary antibodies only (control). Scale bars = 20 µm.

To validate some of the interactions identified through MS, we performed co-immunoprecipitation assays in 293T cells expressing FLAG-tagged wild-type or NS5-_TBEV_NLSm TBEV NS5. To ensure comparable expression levels between the NS5 versions (Fig. 1), cells transfected with NS5-_TBEV_NLSm received four times the amount of plasmid used for wild-type NS5. We selected five high-confidence partners based on the availability of commercial antibodies: ZRANB2, IWS1, SETD2, SMN1, and SCRIB. ZRANB2 functions as a splicing factor (30), while IWS1 is an mRNA splicing and export factor that recruits SETD2 to chromatin to promote histone methylation (31). SETD2 has also been reported to mediate STAT1 methylation in the cytoplasm (32). SMN1 localizes to both the cytoplasm and the nucleus, where it is found in Gems and Cajal bodies (33), whereas SCRIB is primarily found at the cell surface (34). Endogenous SETD2, SCRIB, IWS1, ZRANB2, and SMN1 co-precipitated with wild-type TBEV NS5 in transfected cells (Fig. 5B), confirming the interaction identified by IP-MS in infected cells (Fig. 4D). ZRANB2 and IWS1 showed no or minimal precipitation with NS5-_TBEV_NLSm (Fig. 5B), indicating that these interactions are predominantly nuclear. This aligns with ZRANB2 and IWS1 localization, which is mainly nuclear in both non-infected and infected HMC3 cells (Fig. 6), as previously described in other cellular models (25, 26).

In contrast, SETD2, SMN1, and SCRIB co-precipitated with NS5-_TBEV_NLSm, suggesting these interactions occur, at least in part, in the cytoplasm (Fig. 5B). These findings align with the detection of SCRIB at the plasma membrane of HMC3 cells (Fig. 6), as well as with interactions between SCRIB and a V5-tagged TBEV NS5 at the surface of transfected canine kidney cells (28). This is also consistent with SMN1 localizing in the cytoplasm and nucleus of infected and non-infected HMC3 cells (Fig. 6), as well as in human lung fibroblast MRC5 cells (33). SETD2 exhibited a bright nuclear signal (Fig. 6). However, a dot-like cytoplasmic signal was also detected when the pixel range was adjusted (Fig. S5A). This cytoplasmic signal was not background noise, as no signal was detected in control cells stained only with the secondary antibody under identical imaging settings. This is consistent with previous reports showing that the protein is present in both nuclear and cytosolic fractions of both 786-0 and HEK293T cells (35).

To predict whether the 20 selected putative NS5 TBEV partners preferentially bind the RdRp or MTase domain, we used AlphaPulldown (36, 37). The interactions between the MTase domain and HAX1, ZRANB2, and ZNF687 yielded interface predicted template modeling (iPTM) scores above 0.6, suggesting potential direct interactions (Fig. 5A, lower panel and Table S3). The iPTM score for the interaction between the RdRp domain and TYK2 was the highest among all partners (0.74) (Table S3), consistent with yeast experiments demonstrating a direct interaction (9). Co-immunoprecipitation assays in 293T cells expressing FLAG-tagged versions of the full-length NS5, the RdRp, or the MTase domains were performed. TYK2 served as a control (9). SETD2, SCRIB, IWS1, ZRANB2, and SMN1 co-precipitated with the MTase domain (Fig. 5C). These results align with some of the AlphaPulldown predictions (Fig. 5A, lower panel), supporting the domain-specific nature of these interactions.

To determine whether the interactions between NS5 and these five partners are mediated by RNA, cell extracts were treated with RNase A prior to immunoprecipitation. As positive controls, we used cells expressing tagged versions of IGF2BP2 and IGF2BP3, two RNA-binding proteins known to form RNA-dependent heterodimers (38, 39). As expected (38, 39), RNase A treatment disrupted their interactions (Fig. S5B). Co-immunoprecipitation of SETD2, IWS1, ZRANB2, and SMN1 with NS5 was reduced in RNase A–treated extracts, compared to untreated controls, while interactions with SCRIB and TYK2 remained unaffected (Fig. 5D). The resistance of SCRIB and TYK2 interactions to RNase A treatment is consistent with previous reports of direct protein–protein interactions between NS5 and TYK2 in yeast assays (9, 29) and NS5 and SCRIB in GST pull-down experiments (28). The interaction between NS5 and ZRANB2 was particularly sensitive to RNase A treatment, suggesting that these two proteins interact in an RNA-dependent manner. Since ZRANB2 remained localized in the nucleus during viral infection (Fig. 6) and interacts with NS5 in the nucleus (Fig. 5B), its binding to NS5 is likely mediated by cellular RNAs.

Together, these results showed that NS5 engages in both RNA-dependent and direct protein–protein interactions with host factors, some occurring in the nucleus and others in the cytoplasm.

### Reduced expression of some high-confidence TBEV NS5 partners modulates viral replication

**Fig 7:**
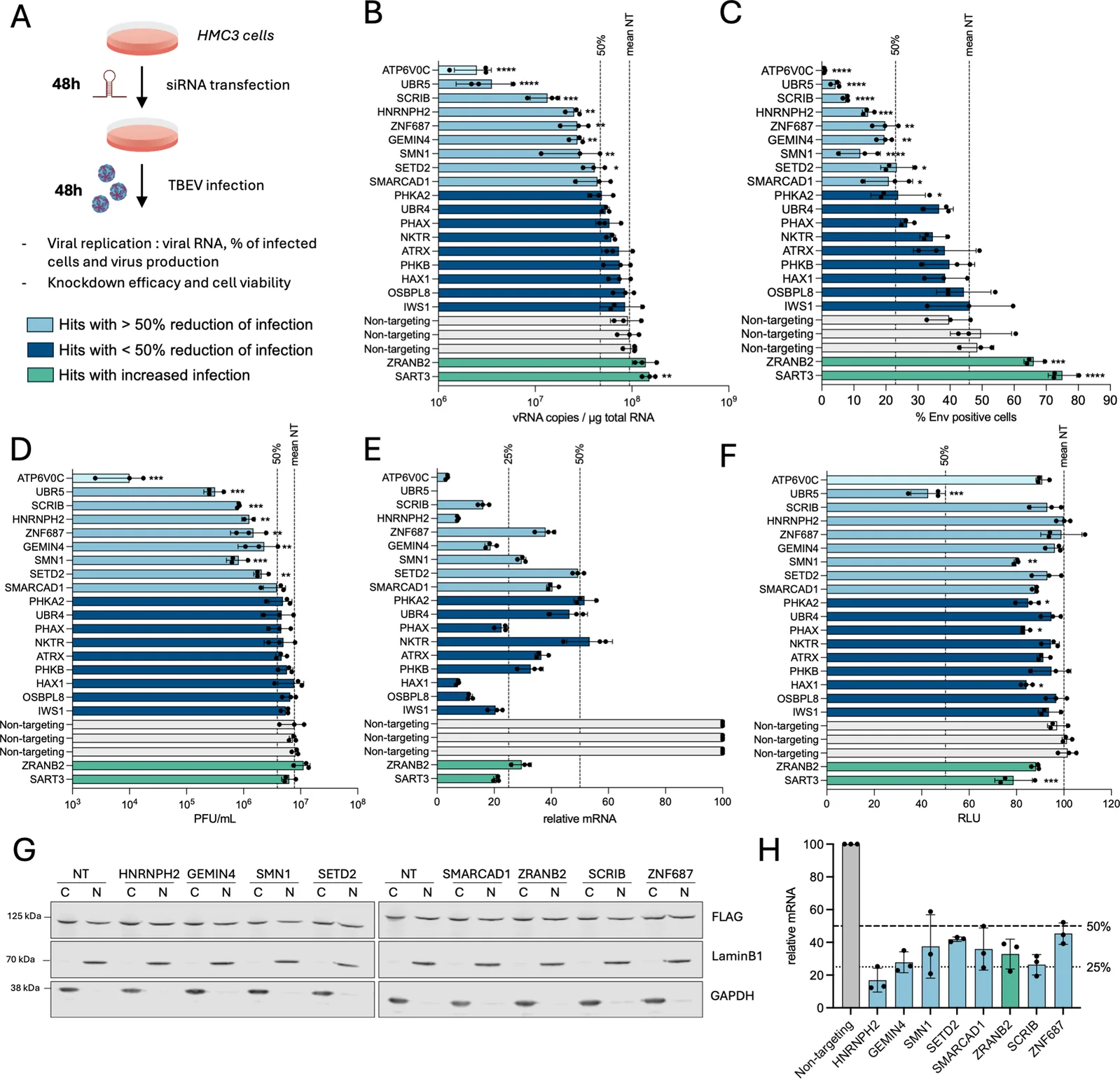
Functional screening of NS5 high-confidence interactants during TBEV infection. (**A**) Scheme summarizing the screen conditions. HMC3 cells were transfected with either a pool of siRNAs against the indicated candidate genes or NT siRNAs and infected with TBEV (at an MOI of 0.001 PFU/cell) for 48 h. (**B**) Cell-associated viral RNA was quantified by RT-qPCR and expressed as vRNA copies/µg of total RNA. (**C**) The percentage of infected cells was assessed by flow cytometry analysis using antibodies against the viral E protein. (**D**) The amount of released infectious virus particles in the supernatants of infected cells was quantified by plaque assays. (**E**) Efficacy of the specific siRNAs. The relative abundances of the mRNAs of the candidate genes were determined by RT-qPCR analysis and were normalized to GAPDH mRNA levels. Values are expressed relative to abundance in cells transfected with NT siRNAs in each experiment, set as 100. (**F**) Cell viability of siRNA-transfected cells was assessed using CellTiter-Glo Luminescent Cell Viability Assays. Data are means ± SD of three independent experiments, *P < 0.05, **P < 0.01, ***P < 0.001, ****P < 0.001, one-way ANOVA test. (**G**) 293T cells were transfected with either a pool of siRNAs against the indicated candidate genes or NT siRNAs for 48 h and were transfected with plasmids encoding TBEV FLAG-NS5. After 24 hours, NS5 subcellular localization was determined by cellular fractionation. Cytoplasmic (C) and nuclear (N) fractions were analyzed by Western blot using antibodies against FLAG, GAPDH, and Lamin B1. (**H**) Efficacy of the specific siRNAs. The relative abundances of the mRNAs of the candidate genes were determined by RT-qPCR analysis and were normalized to GAPDH mRNA levels. Values are expressed relative to abundance in cells transfected with NT siRNAs in each experiment, set as 100.

To assess whether the 19 high-confidence TBEV NS5 partners that we selected (Fig. 5A) impact viral replication, a loss-of-function screen was performed (Fig. 7A). HMC3 cells were transfected with pools of four siRNAs targeting the selected NS5 interactants and infected 48 h later with TBEV at an MOI of 0.001. siRNAs targeting ATP6V0C, a protein promoting fusion between the endosomal membrane and the envelope of orthoflaviviruses (40), were used as positive controls. Three sets of non-targeting (NT) siRNA pools were used as negative controls. At 48 h post-infection, intracellular viral RNA levels were evaluated by RT-qPCR analysis (Fig. 7B), and the number of cells positive for the viral protein E was quantified by flow cytometry analysis (Fig. 7C). Supernatants were also harvested and incubated on Vero cells to evaluate infectious viral particle production (Fig. 7D). cDNA samples were used to analyze mRNA levels of the selected genes and assess knock-down efficiency (Fig. 7E). Finally, cell viability was measured at 72 h post-transfection in mock-infected samples (Fig. 7F). Viral replication measurements were compared to those obtained from cells transfected with the non-targeting siRNA pool. As expected (40), reducing ATP6V0C significantly decreased viral RNA yield, viral protein expression, and viral particle production without affecting cell viability (Fig. 7B through F). Reduced expression of eight genes (*UBR5, SCRIB, HNRNPH2, ZNF687, GEMIN4, SMN1, SETD2,* and *SMARCAD1*) decreased viral RNA yield, the number of infected cells, and/or viral titers by over 50% compared with cells transfected with control siRNAs (Fig. 7B through D). Reduced expression of nine NS5 partners had little to no effect on TBEV replication (Fig. 7B through D). ZRANB2 and SART3, two RNA-binding proteins involved in the regulation of mRNA splicing (30, 41–43)*,* behaved like antiviral genes since knocking down their expression significantly increased viral RNA yield and/or the percentage of infected cells (Fig. 7B through D). RT-qPCR analyses revealed that all the siRNA pools were reducing the expression of their respective targets by at least 50% (Fig. 7E). The viability of cells treated with siRNAs against UBR5 and SART3 decreased by over 20% compared to control cells (Fig. 7F). Therefore, data related to UBR5 and SART3 silencing should be interpreted with caution.

To test whether any of the NS5 cellular partners affected its nuclear trafficking, we selected those whose reduced expression modulated viral replication in at least one of the three virological assays without affecting cell viability: SCRIB, HNRNPH2, ZNF687, GEMIN4, SMN1, SETD2, SMARCAD1, and ZRANB2 (Fig. 7B through F). Experiments were performed in 293T cells transfected with siRNAs targeting these eight factors. At 48 h post-transfection, cells were transfected with FLAG-tagged NS5 for 24 h and then subjected to subcellular fractionation (Fig. 7G). The efficiency of the siRNA-mediated knockdown was assessed by RT-qPCR (Fig. 7H), confirming the results obtained in HMC3 cells (Fig. 7E). Despite the reduced expression of these genes (Fig. 7H), the distribution of NS5 between the nuclear and cytosolic fractions remained unchanged (Fig. 7G). These findings suggest that these proteins do not influence NS5 nuclear trafficking.

Together, our data show that seven NS5 partners that are known to have nuclear activities (HNRNPH2, ZNF687, GEMIN4, SMN1, SETD2, SMARCAD1, and ZRANB2) significantly modulated viral replication without affecting NS5 localization, further suggesting that nuclear NS5 plays a role in the viral life cycle.

## DISCUSSION

Since NS5 is too large to passively diffuse between the nucleus and the cytoplasm, it relies on active transport through the nuclear pore for nuclear import. We identified three residues, K389, K391, and R393, within the α/βNLS that are essential for the nuclear import of TBEV NS5. Similar findings have been reported for mosquito-borne orthoflaviviruses, where K390 and R393 in ZIKV, the REE motif (residues 396–398) in DENV2, and the REK motif (residues 389–393) in WNV play pivotal roles in NS5 nuclear localization (10, 12, 44). Thus, the α/βNLS appears to be a conserved primary determinant of NS5 nuclear addressing across orthoflaviviruses. Notably, DENV2 also possesses a C-terminal NLS, indicating virus-specific features within the orthoflavivirus genus (45). While deletion of the β1NLS domain impaired NS5 nuclear localization, mutation of its positively charged residues did not, suggesting that the deletion may induce conformational changes that indirectly disrupt nuclear import. Although we did not identify β-importin1 or α-importin proteins as cellular partners of TBEV NS5, we found IPO4, IPO7, and IPO9, which are members of the importin family, as specific interactors. Future studies could involve reducing the expression of these proteins to assess their role in NS5 nuclear import. We also identified CRM1 (also called Exportin-1 [XPO1]) as a cellular partner of TBEV NS5, as previously shown for DENV NS5 (46), suggesting that NS5 may actively shuttle between the cytoplasm and the nucleus. However, our results indicate that the distribution of NS5 WT and NS5-_TBEV_NLSm was unaffected by LMB, suggesting that TBEV NS5 is exported from the nucleus in a CRM1-independent manner. This finding was surprising since CRM1-dependent export has been proposed as a common feature of flavivirus NS5 proteins. Dependency on CRM1 was suggested for both DENV and WNV NS5 nuclear export in Vero cells treated with LMB (46, 47). As for other flaviviruses, including ZIKV and JEV, CRM1 involvement was only inferred from sequence conservation (46) rather than experimentally demonstrated. Furthermore, the claim of a highly conserved CRM1-dependent NES should be interpreted with caution. The residues identified as critical for CRM1-dependent export in DENV NS5 include the hydrophobic motif IIxxV, whereas the corresponding sequence in TBEV NS5 is ARxxV. The substitution of isoleucine by arginine represents a non-conservative change from a hydrophobic to a positively charged residue. Given that classical CRM1-dependent NES motifs rely on exposed hydrophobic residues for CRM1 recognition, this difference raises the possibility that the corresponding motif in DENV and TBEV is not functionally equivalent. The identification of CRM1 among NS5-associated proteins highlights that MS identifies proteins present within the same molecular complex but does not establish the nature, directness, or functional consequence of the interaction. The mechanisms underlying TBEV NS5 nuclear trafficking thus warrant further investigation.

Previous studies have suggested that shuttling between the cytoplasm and the nucleus protects ZIKV NS5 from degradation (11). A similar mechanism may apply to TBEV, as NS5-_TBEV_NLSm was less expressed than wild-type NS5, though to a lesser extent than for ZIKV NS5. Our CHX and MG132 results further suggest that NS5-NLSm is less stable than the wild-type protein and indicate that it may be degraded via the proteasome. Alternatively, the wild-type protein may intrinsically be more stable than the mutant, independently of its localization. Mutation of key residues for NS5 nuclear import abolished viral RNA replication of a TBEV replicon. While these mutations do not lie within the catalytic pocket of the RdRp domain, they could induce conformational changes that indirectly impair replication of the NLSm. In the case of DENV, ZIKV, and WNV, RdRp activity of the NS5 NLSm showed only marginal, if any, impact on the polymerase function (10, 11, 47), suggesting that defective replication is not due to a loss of enzymatic activity. However, the absence of RNA replication, even at very early stages, suggests that additional factors might be at play, like reduced interactions between NS5 and viral or host proteins required for replication. In DENV, viral replication was unaffected by NS5 mutants with reduced nuclear localization, suggesting that the extent of nuclear import is not directly correlated with replication efficiency (10). Importin inhibitors used to block NS5 nuclear import in DENV- and ZIKV-infected cells significantly reduced infection (48–50). However, their broad effect on the cells prevents definitive conclusions about the specific role of nuclear NS5 in viral replication. Assessing the significance of NS5 nuclear localization in an infectious context remains challenging, as both mutational and pharmacological inhibition approaches may introduce indirect effects.

Previous interactomic studies of orthoflavivirus proteins have relied on tagged versions of proteins expressed individually (16, 17) or from modified infectious clones (14). Here, we used NS5 antibodies to immunoprecipitate NS5 and its partners in infected 293T or HMC3 cells. Both AP- and IP-MS methodologies have drawbacks. For IP, it is possible that epitopes bound by antibodies used for precipitation will mask interactions with cellular partners. We used a polyclonal antibody mixture, which can mitigate these effects by targeting multiple epitopes, but it is still possible that predominant epitopes may mask interactions. AP methods require the introduction of tags to proteins that can disrupt protein structure and impact cellular interaction partners. Proteins expressed with tags may not localize properly, and it is very difficult to introduce tags into flavivirus genomes without significantly impacting infectivity. Using the two approaches in parallel could be informative. Indeed, comparing the NS5 partners we identified previously by immunoprecipitation of FLAG-tagged NS5 expressed in 293T cells (9) with those recovered here by IP-MS revealed limited overlap, with only 35 common partners. This divergence may be attributed to the use of different antibodies. Immunoprecipitation via the FLAG tag offers the advantage of better-controlled background noise, as associated contaminants are well-characterized. In contrast, the use of anti-NS5 antibodies, while enabling analysis in the context of infection, may introduce higher background noise. However, the inclusion of non-infected cells as controls helps mitigate this effect. Additionally, differences in the lysis buffers used in the two protocols may influence the profile of detected interactions, particularly impacting indirect interactions and multiprotein complexes. Among the 35 partners identified by both approaches are TYK2, ATRX, SMRCD, and members of the GEMIN complex. These proteins were also identified as partners of POWV NS5 through AP-MS in 293T and HMC3 cells expressing Strep-tagged NS5 proteins (Molho et al., unpublished).

We validated the interactions between SETD2, SCRIB, IWS1, ZRANB2, and SMN1 with NS5 by co-immunoprecipitation. The binding of SETD2, IWS1, and SMN1 to NS5 was reduced in transfected cell extracts treated with RNase A, suggesting that cellular RNA may stabilize these interactions. The interaction between NS5 and ZRANB2 was highly sensitive to RNase A treatment, indicating that these two proteins require RNA for their association. Indeed, the potential binding of NS5 to cellular RNAs has not been explored. To identify potential cellular RNAs bound to NS5, one approach could involve immunoprecipitation of NS5, either from transfected or infected cells, followed by RNA extraction and RNA-seq analysis.

The strong enrichment of nuclear proteins among NS5-associated cellular factors suggests that NS5 may have multiple functions in the nucleus. We identified six NS5 partners with described nuclear activities (HNRNPH2, ZNF687, GEMIN4, SMN1, SETD2, and SMARCAD1) that act as proviral factors for TBEV replication. GEMIN4, SMN1, HNRNPH2, and ZNF687 are all involved in splicing processes, while SETD2 and SMARCAD1 participate in chromatin regulation. Similar to DENV NS5, TBEV NS5 may co-opt these proteins to modulate host splicing and gene expression, thereby suppressing antiviral responses and promoting replication (14, 17, 51). Interestingly, the interaction of the cytoplasmic NS5 mutant with SETD2 and SMN1 suggests that these interactions occur, at least partly, in the cytoplasm. This raises the possibility that NS5 alters the subcellular distribution of these proteins, preventing them from performing their nuclear function. However, we did not detect a relocalization of these proteins during infection. Functional consequences of protein–protein interactions may include modulation of enzymatic activity, inhibition of signaling pathways, regulation of post-translational modifications, or disruption of molecular complexes, without triggering detectable changes in steady-state localization. For instance, TBEV NS5 interacts with TYK2 and inhibits its kinase activity without affecting its localization (9). A dynamic interaction with NS5 may also impact other interactions of these proteins and thereby their function. Future work will enable us to disentangle how this may operate.

Finally, we identified ZRANB2 as an antiviral nuclear partner of TBEV NS5. ZRANB2 is an RNA-binding protein involved in pre-mRNA splicing (30, 41, 42). It also interacts with the NS5 protein of WNV (16), DENV, ZIKV (17), and JEV (15) and inhibits the replication of WNV, DENV, and ZIKV (16). Interestingly, its phosphorylation level increases during DENV and WNV infections (52, 53). These findings suggest that ZRANB2 is a conserved nuclear partner of orthoflaviviruses NS5 and may play an antiviral role in their replication. Our data showed that it interacts with the MTAse domain of TBEV NS5, predominantly in the nucleus of infected cells, and in an RNA-dependent manner. Further characterization of the interaction between NS5 and ZRANB2 could help determine its function during viral replication. Additionally, analyzing the spliceosome composition in infected cells, with or without ZRANB2, may reveal changes in the expression of host factors critical for viral replication.

Importantly, some cellular partners that did not appear to modulate viral infection in our functional assay may still play critical roles in the viral life cycle. This limitation arises because our assays were constrained by several factors, including the use of a single cell type, a unique time point, and an *in vitro* environment that does not fully recapitulate the complexity of viral infection *in vivo*. Further investigations into the nuclear activities of TBEV NS5 are therefore essential.

## MATERIALS AND METHODS

### Cells and viruses

Human embryonic kidney (HEK) 293T cells and Human Microglial Clone 3 (HMC3) cells were obtained from the American Type Culture Collections (ATCC, CRL-3126 and CRL-3304). Human hepatocellular carcinoma cells (Huh7) were kindly provided by A. Martin, Institut Pasteur Paris. African green monkey kidney epithelial Vero cells were kindly provided by F. Tangy, Institut Pasteur Paris. All cultures were grown under standard conditions in Dulbecco’s Modified Eagle Medium containing GlutaMAX (Gibco) and supplemented with 10% heat-inactivated fetal bovine serum (FBS), and 1% penicillin and streptomycin (10,000 IU/mL; Thermo Fisher Scientific).

Experiments with TBEV were performed in a BSL-3 laboratory, following safety and security protocols approved by the risk prevention service of Institut Pasteur. The TBEV strain (Hypr strain, isolated in the Czech Republic in 1953) was obtained from the European Virus Archive (EVAg, 008v-EVA285). Virus stocks were produced on Vero cells. Titration of infectious virions was performed by plaque assay on Vero cells, as previously described for other orthoflaviviruses (54). HMC3 and 293T cells were infected at the MOI indicated in the figure legends for 2 h in DMEM medium containing 2% FBS, followed by replacement with 10% FBS media.

### Antibodies and reagents

Anti-TBEV NS5 antibodies were obtained by immunization of a rabbit using full-length recombinant TBEV NS5, as previously described (9). They were used at a dilution of 1:1,000 for Western blot and immunofluorescence assays. Anti-E Mab 4G2 pan-orthoflavivirus antibody (kind gift from Philippe Desprès, PIMIT, Reunion Island) was used at a dilution of 1:1,000 for flow cytometry. For immunofluorescence assays, either the 4G2 or the anti-TBEV-E A1 (kind gift from Karin Stiasny and Judith Aberle, Medical University of Vienna) was used. Anti-FLAG M2 (F3165, Sigma-Aldrich) was used at a dilution of 1:5,000 for Western blot. Anti-lamin B1 (12987-1-AP, Proteintech) and anti-GAPDH (10494-1-AP, Proteintech) were used at a dilution of 1:5,000 and 1:10,000 for Western blot, respectively. Anti-IWS1 (#5681, Cell Signaling Technology), anti-SETD2 (#80290, Cell Signaling Technology), anti-Scribble (#4475, Cell Signaling Technology), anti-SMN1 (#12976, Cell Signaling Technology), anti-ZRANB2 (24816-1-AP, Proteintech), and anti-TYK2 antibodies (9) were used at a dilution of 1:1,000 for Western blot and 1:200 for immunofluorescence. The following secondary antibodies were used: Alexa Fluor 488 goat anti-mouse IgG (H+L) (A11001, Invitrogen); Alexa Fluor 647 donkey anti-mouse IgG (H+L) (A31571, Invitrogen); Alexa Fluor 647 goat anti-rabbit IgG (H+L) (A21244, Life Technologies); Alexa Fluor 680 goat anti-mouse IgG (H+L) (A21058, Invitrogen); and goat anti-rabbit IgG (H+L) Dylight 800 (SA5-35571, Invitrogen). All secondary antibodies were used at a dilution of 1:1,000 for flow cytometry and immunofluorescence assays and 1:10,000 for Western blot analysis. Leptomycin B was diluted in methanol to a final concentration of 10 nM (Sigma-Aldrich L2913). CHX (Sigma-Aldrich, C4859) and MG132 (Sigma-Aldrich, M7449) were diluted in DMSO to final concentrations of 100 µg/mL and 10 µM, respectively. Methanol or DMSO alone were used as negative controls for these experiments.

### Plasmids and cloning

Plasmids coding for full-length, MTase and RdRp domains of TBEV NS5, as well as for TBEV C, NS2B and ZIKV NS5, in p3xFLAG-CMV10 (Sigma) were previously described (9). Deletion mutants of TBEV NS5 were generated by PCR using p3xFLAG-WT-TBEV-NS5 as a template and the primers listed in Table S4. Point mutants of TBEV and ZIKV NS5 were generated by site-directed mutagenesis of p3xFLAG-WT-NS5 or TBEV replicon using the primers listed in Table S4 and the Phusion Site-Directed Mutagenesis Kit (10493438, Fisher Scientific), following the manufacturer’s instructions. Plasmids encoding IGF2BP1 and IGF2BP3 were from Eugene Yeo’s and Thomas Tuschl’s laboratories, respectively, and were acquired through Addgene (#155676 and #19879). pCSC-GFP-NES-EGFP was a gift from Baojin Ding (Addgene plasmid #221810; https://www.addgene.org/221810/; RRID:Addgene_221810). All plasmids were amplified in TOP10 cells (Thermo Fisher Scientific), and sequencing was outsourced to Eurofins Genomics.

### TBEV replicons

WT and GAA replication-defective TBEV replicons were previously described (9). TBEV pTND/ΔME replicon plasmids (24) were linearized by NheI digestion and blunt-ended using Quick Blunting Kit (New England Biolabs). Five micrograms of purified DNA template was used for T7 *in vitro* transcription using RiboMAX large-scale RNA production system T7 (Promega) in the presence of 40 mM cap analog (Ribo m7G Cap, Promega) following the manufacturer’s instructions. After RQ1 DNase treatment (Promega), RNA was purified with an RNA clean-up kit (Macherey-Nagel). *In vitro* synthesized TBEV replicon RNA was introduced into HMC3 cells by electroporation. Briefly, 2.5 × 10^6^ trypsinized HMC3 cells were washed three times in cold PBS and resuspended in 400 µL cold PBS and electroporated with 2.5 µg of RNA in 0.4 cm electroporation cuvettes (Bio-Rad) with a 25 µF and 450 V pulse using the GenePulser system (Bio-Rad). After electroporation, cells were collected in 12 mL of warm medium, the cell suspension was transferred to 12-well plates (1 mL per well), and incubated at 37°C under standard conditions.

### Plasmid transfections

293T and HMC3 cells were transfected using TransIT-293 (Mirus) and FuGENE HD (Promega) transfection reagents, respectively, following the manufacturer’s protocol. One day later, samples were processed for Western blot and co-immunoprecipitation.

### Western blot

Cells were lysed in radio-immunoprecipitation assay (RIPA) buffer (Sigma-Aldrich) supplemented with a protease inhibitor cocktail (Roche) for 15 min at 4°C, and lysates were clarified by centrifugation at 10,000 × *g* at 4°C for 15 min. Samples were denatured at 95°C in 1× Protein Sample Loading Buffer (Li-Cor Bioscience) under reducing conditions (NuPAGE reducing agent, Thermo Fisher Scientific). Proteins were separated by SDS-PAGE using NuPage 4%–12% Bis-Tris gels (Invitrogen) and transferred to nitrocellulose membranes (Bio-Rad) using a Trans-Blot Turbo Transfer system (Bio-Rad). Alternatively, high molecular weight proteins were separated by SDS-PAGE using NuPAGE 3-8% Tris-Acetate gels (Invitrogen) and were transferred to nitrocellulose membranes using liquid transfer buffer (50 mM Tris, 25 mM glycine, 0.4% SDS, and 20% ethanol) for 2 h at 25 V. Membranes were blocked with PBS-0.1% Tween 20 (PBS-T) containing 5% milk and incubated overnight at 4°C with primary antibodies diluted in blocking buffer. Finally, membranes were washed and incubated for 45 min at room temperature with secondary antibodies diluted in blocking buffer and washed again. Images were acquired using an Odyssey CLx infrared imaging system (Li-Cor Bioscience).

### Co-immunoprecipitation

293T cells were transfected with FLAG-tagged NS5 plasmids for 24 h and were lysed in IP lysis buffer (50 mM Tris-HCl pH 7.5, 150 mM NaCl, 1 mM EDTA, Complete protease inhibitor tablet (EDTA-free), and 0.5% IGEPAL CA-630) for 30 min at 4°C. Lysates were clarified by centrifugation at 5,000 × *g* for 20 min at 4°C. Clarified lysates were incubated with magnetic beads coupled with anti-FLAG M2 (M8823; Sigma-Aldrich) or anti-V5 (SAE0203; Sigma-Aldrich) overnight at 4°C. Following incubation, beads were washed four times with IP washing buffer (50 mM Tris-HCl, pH 7.5, 150 mM NaCl, 1 mM EDTA, and 0.05% IGEPAL CA-630), then once with detergent-free IP buffer (50 mM Tris-HCl, pH 7.5, 150 mM NaCl, and 1 mM EDTA), and proteins were eluted with 1× Protein Sample Loading Buffer (Li-Cor Bioscience) and 1× NuPAGE reducing agent (Thermo Fisher Scientific). For nuclease experiments, clarified lysates were treated with 100 µg/mL of RNase A (EN0531; Thermo Fisher Scientific) for 20 min at room temperature prior to immunoprecipitation. Proteins of interest were detected by Western blot, as described above.

### Cellular fractionation

The protocol was adapted from reference 55. 293T cells were harvested and washed once with ice-cold PBS. After 5 min of centrifugation at 300 × *g*, cells were lysed with low salt lysis buffer (10 mM Tris, pH 7.5, 10 mM NaCl, 3 mM MgCl2, 0.3% IGEPAL CA-630, and 10% glycerol) for 10 min on ice to preserve nuclear integrity. After centrifugation at 800 × *g* for 8 min at 4°C, the supernatant was saved for the “cytoplasmic” fraction. The pellet containing the nuclei was washed four times with low salt lysis buffer by centrifugation at 300 × *g* for 4 min at 4°C. Finally, the pellets containing washed nuclei were lysed with a high salt denaturing buffer (10 mM Tris-HCl, pH 7.0, 4 mM EDTA, 0.3 M NaCl, 1 M urea, and 1% IGEPAL CA-630) to generate nuclear extracts containing solubilized nuclear proteins and protein stably bound to the chromatin.

### Immunofluorescence assays

HMC3 and Huh7 cells were plated into 24-well plates on glass coverslips. After transfection or infection, wells were fixed with 4% paraformaldehyde (PFA) (Sigma-Aldrich) for 30 min at room temperature, permeabilized with 50% vol/vol methanol/ethanol (Sigma-Aldrich) vol/vol for 15 min, and then blocked for 30 min with PBS containing 0.05% Tween and 5% BSA before incubation with the indicated primary antibodies for 1 h. After incubation, cells were washed three times with PBS containing 0.05% Tween. Secondary Alexa Fluor 488- or 647-conjugated antibodies and DAPI were added for 1 h, then cells were washed three times with PBS containing 0.05% Tween. Slides were mounted using Prolong Gold (Life Technologies, P36930) imaging medium. Images were acquired using a Zeiss LSM700 confocal microscope or a Zeiss Imager Z1 epifluorescence microscope. Quantification of the nuclear localization of NS5 WT or mutants was determined by quantifying the intensity of cytoplasmic (C) and nuclear (N) FLAG signal using Fiji, followed by calculation of the ratio of mean fluorescence N/C.

### Flow cytometry

Infected cells were harvested using trypsin and fixed with cytofix/cytoperm kit (BD, Pharmingen). Cells were washed twice with Perm/Wash buffer (BD, Pharmingen) and stained using the anti-E Mab 4G2 primary antibody diluted in Perm/Wash buffer for 1 h at 4°C. Cells were washed twice and stained with secondary anti-mouse Alexa 647 in the dark for 45 min at 4°C. After washing the cells twice, data were acquired using the Attune NxT Acoustic Focusing Cytometer (Life Technologies) and analyzed using FlowJo software.

### RNA extraction and RT-qPCR analyses

Total RNAs from cells were extracted using the NucleoMag RNA kit (Macherey-Nagel) following the manufacturer’s protocol. First-strand cDNA synthesis was performed on 1 µg of total RNA with the RevertAid H Minus Moloney murine Leukemia Virus (M-MuLV) reverse transcriptase (Thermo Fisher Scientific) using random primers p(dN)_6_ (Roche). Quantitative real-time PCR was performed on a real-time PCR system (Quant Studio 6 Flex; Applied Biosystems) with SYBR green PCR master mix (Life Technologies). Data were analyzed by the ΔΔCt method, with all samples normalized to GAPDH. All experiments were performed in technical triplicate. The primers used for RT-qPCR are listed in Table S5. Quantification of TBEV genomes was determined by extrapolation from a standard curve generated from serial dilutions of the plasmid encoding TBEV NS5 (p3xFLAG-NS5-TBEV).

### Sample preparation for MS

HMC3 and 293T cells were infected with TBEV for 24 h, then harvested by trypsinization and counted. In all, 500,000 cells were used for flow cytometry staining of the viral envelope to check the efficacy of infection. Non-infected cells were used as controls. For affinity purification of NS5, 10 million cells were lysed in 1 mL of IP lysis buffer (50 mM Tris-HCl, pH 7.5, 150 mM NaCl, 1 mM EDTA, Complete protease inhibitor tablet [EDTA-free], and 0.5% IGEPAL CA-630) for 30 min at 4°C. Lysates were clarified by centrifugation at 5,000 ×*g* for 20 min at 4°C. Clarified lysates were incubated with 10 µg of anti-TBEV NS5 antibody overnight at 4°C. Following incubation, 40 µL of Pierce Protein A/G magnetic beads (#88802, Thermo Fisher Scientific) were added to the lysate–antibody mixture and incubated for 2 h at 4°C. Beads were washed four times with IP washing buffer (50 mM Tris-HCl, pH 7.5, 150 mM NaCl, 1 mM EDTA, and 0.05% IGEPAL CA-630). Three microliters of beads was eluted with 1× Protein Sample Loading Buffer (Li-Cor Bioscience) and 1× NuPAGE reducing agent (Thermo Fisher Scientific). The eluate was analyzed by SDS-PAGE and silver staining (Pierce). For MS, immunoprecipitates were directly digested on beads. Beads were washed once and resuspended with 40 µL of 2 M urea, 50 mM Tris, pH 8.0, and 1 mM DTT, and incubated for 30 min at 37°C with shaking at 700 rpm. Iodoacetamide (Sigma-Aldrich) was added to 3 mM final concentration and incubated at room temperature for 45 min in the dark with shaking. Digestion was performed by adding 3 mM DTT and 750 ng of sequencing grade modified trypsin (Promega) and incubating at 37°C with shaking overnight. Finally, supernatants were collected to fresh tubes and an additional 500 ng of sequencing grade modified trypsin was added and incubated for 2 h at 37°C with shaking. Digested samples were desalted using C18 solid phase extraction columns (C18 BioPurSPN, Nest Group) according to the manufacturer’s instructions. Desalted samples were vacuum centrifuged to dryness and resuspended in 0.1% formic acid for mass spectrometry analysis.

### Mass spectrometry and data analyses

All samples were analyzed on an Orbitrap Eclipse mass spectrometry system equipped with an Easy nLC 1200 ultra-high pressure liquid chromatography system interfaced via a Nanospray Flex nanoelectrospray source (Thermo Fisher Scientific). Samples were injected onto a fritted fused silica capillary (30 cm × 75 µm inner diameter with a 15 μm tip, CoAnn Technologies) packed with ReprosilPur C18-AQ 1.9 μm particles (Dr. Maisch GmbH). Buffer A consisted of 0.1% formic acid in water, and buffer B consisted of 0.1% formic acid in 80% acetonitrile. Peptides were separated by an organic gradient from 5% to 35% mobile buffer B over 60 min, followed by an increase to 100% B over 10 min at a flow rate of 300 nL/min. Analytical columns were equilibrated with 3 μL of buffer A.

To build a spectral library, samples from each set of biological replicates were pooled and acquired in a data-dependent manner. Data-dependent acquisition (DDA) was performed by acquiring a full scan over a m/z range of 375–1,025 in the Orbitrap at 120,000 resolving power (@ 200 m/z) with a normalized AGC target of 100%, an RF lens setting of 30%, and an instrument-controlled ion injection time. Dynamic exclusion was set to 30 seconds, with a 10 p.p.m. exclusion width setting. Peptides with charge states 2–6 were selected for MS/MS interrogation using higher energy collisional dissociation (HCD) with a normalized HCD collision energy of 28%, with 3 s of MS/MS scans per cycle. Data-independent analysis (DIA) was performed on all individual samples. A full scan was collected at 60,000 resolving power over a scan range of 390–1,010 m/z, an instrument-controlled AGC target, an RF lens setting of 30%, and an instrument-controlled maximum injection time, followed by DIA scans using 8 m/z isolation windows over 400–1,000 m/z at a normalized HCD collision energy of 28%.

The Spectronaut algorithm was used to build spectral libraries from DDA data, identify peptides/proteins, and extract intensity information from DIA data (56). DDA data were searched against the *Homo sapiens* UniProt reference proteome (downloaded on 23 August 2023) and TBEV protein sequences. False discovery rates were estimated using a decoy database strategy. All data were filtered to achieve a false discovery rate of 0.01 for peptide-spectrum matches, peptide identifications, and protein identifications. Search parameters included a fixed modification for carbamidomethyl cysteine and variable modifications for N-terminal protein acetylation and methionine oxidation. All other search parameters were Biognosys factory defaults.

Statistical analysis of proteomics data was conducted utilizing the MSstats package in R (57) and SAINTq for interaction scoring (58). For MSstats analysis, all data were normalized by equalizing median intensities, the summary method was Tukey’s median polish, and the maximum quantile for deciding censored missing values was 0.999. The interaction network of TBEV NS5 protein was represented using Cytoscape software (v3.9.1). Host protein–protein interactions and Gene Ontology analysis were performed using stringApp on Cytoscape (https://apps.cytoscape.org/apps/stringapp).

### siRNA screening

Pools of four siRNAs for each selected host target (Horizon Discovery) were transfected in HMC3 cells using HiPerFect (Qiagen) transfection reagent according to the manufacturer’s recommendations at a final siRNA concentration of 10 nM. After 48 h of transfection, cells were infected with TBEV at an MOI of 0.001. At 48 h post-infection, viral replication was assessed by measuring the percentage of infected cells by flow cytometry, cell-associated viral RNA by RT-qPCR, and the amount of released infectious virus particles in the supernatants by plaque assays. Cytotoxicity of siRNAs was assessed at 72 h post-transfection using the CellTiter-Glo Luminescent Cell Viability Assay (Promega) according to the manufacturer’s instructions.

### Computational interaction analysis using AlphaFold 3

Computational predictions of protein–protein interactions were generated using AlphaFold 3 (AF3) (37). All candidate sequences were retrieved from UniProt, and for TBEV-NS5, we used residues 1–255 for the MTase domain and 276–875 for the RdRp domain. AF3 was run with default parameters for multimolecular complex prediction on the AlphaFold Server (https://alphafoldserver.com), modeling each complex as a single 1:1 assembly. For each interaction, the average of five models was used, selected according to AF3 confidence metrics, including per-residue confidence and interface confidence (iPTM).

### Data representation and statistical analyses

Data are presented and analyzed using GraphPad Prism 10. Significance was calculated as indicated in the corresponding figure legend. Where not indicated, no statistical significance was determined.

## Data Availability

All data necessary to evaluate the conclusions in this study are provided in the main text and the supplemental material. The mass spectrometry proteomics data have been deposited to the ProteomeXchange Consortium via the PRIDE (59) partner repository with the dataset identifier PXD080823.

## SUPPLEMENTAL MATERIAL

Supplemental figures jvi.00810-26-s0001.pdf Fig. S1 to S5.

Supplemental legends jvi.00810-26-s0002.docx Legends for Fig. S1 to S5.

Table S1 jvi.00810-26-s0003.xlsx SAINTq analysis of TBEV NS5 DIA-MS data.

Table S2 jvi.00810-26-s0004.xlsx Global proteomics of TBEV-infected cells.

Table S3 jvi.00810-26-s0005.xlsx AlphaFold Pulldown TBEV NS5 and high-confidence partners.

Table S4 jvi.00810-26-s0006.xlsx Primers for cloning.

Table S5 jvi.00810-26-s0007.xlsx Primers for qPCR.

ASM does not own the copyrights to Supplemental Material that may be linked to, or accessed through, an article. The authors have granted ASM a non-exclusive, world-wide license to publish the Supplemental Material files. Please contact the corresponding author directly for reuse.

## References

[1] Pierson TC, Diamond MS. 2020. The continued threat of emerging flaviviruses. Nat Microbiol 5:796–812. doi:10.1038/s41564-020-0714-0 PMC7696730 32367055

[2] Gubler DJ. 2007. The continuing spread of West Nile virus in the western hemisphere. Clin Infect Dis 45:1039–1046. doi:10.1086/521911 17879923

[3] Weaver SC, Costa F, Garcia-Blanco MA, Ko AI, Ribeiro GS, Saade G, Shi P-Y, Vasilakis N. 2016. Zika virus: history, emergence, biology, and prospects for control. Antiviral Res 130:69–80. doi:10.1016/j.antiviral.2016.03.010 PMC4851879 26996139

[4] Venkatesan P. 2024. Global upsurge in dengue in 2024. Lancet Infect Dis 24:e620. doi:10.1016/S1473-3099(24)00609-1 39341223

[5] Douam F, Ploss A. 2018. Yellow fever virus: knowledge gaps impeding the fight against an old foe. Trends Microbiol 26:913–928. doi:10.1016/j.tim.2018.05.012 PMC6340642 29933925

[6] Campbell O, Krause PJ. 2020. The emergence of human Powassan virus infection in North America. Ticks Tick Borne Dis 11:101540. doi:10.1016/j.ttbdis.2020.101540 32993949

[7] Goh JZH, De Hayr L, Khromykh AA, Slonchak A. 2024. The Flavivirus non-structural protein 5 (NS5): structure, functions, and targeting for development of vaccines and therapeutics. Vaccines (Basel) 12:865. doi:10.3390/vaccines12080865 PMC11360482 39203991

[8] Best SM. 2017. The many faces of the Flavivirus NS5 protein in antagonism of type I interferon signaling. J Virol 91:e01970-16. doi:10.1128/JVI.01970-16 PMC5244349 27881649

[9] Gracias S, Chazal M, Decombe A, Unterfinger Y, Sogues A, Pruvost L, Robert V, Lacour SA, Lemasson M, Sourisseau M, Li Z, Richardson J, Pellegrini S, Decroly E, Caval V, Jouvenet N. 2023. Tick-borne flavivirus NS5 antagonizes interferon signaling by inhibiting the catalytic activity of TYK2. EMBO Rep 24:e57424. doi:10.15252/embr.202357424 PMC10702846 37860832

[10] Kumar A, Bühler S, Selisko B, Davidson A, Mulder K, Canard B, Miller S, Bartenschlager R. 2013. Nuclear localization of dengue virus nonstructural protein 5 does not strictly correlate with efficient viral RNA replication and inhibition of type I interferon signaling. J Virol 87:4545–4557. doi:10.1128/JVI.03083-12 PMC3624364 23408610

[11] Ji W, Luo G. 2020. Zika virus NS5 nuclear accumulation is protective of protein degradation and is required for viral RNA replication. Virology 541:124–135. doi:10.1016/j.virol.2019.10.010 32056710

[12] Ng IHW, Chan KW-K, Tan MJA, Gwee CP, Smith KM, Jeffress SJ, Saw W-G, Swarbrick CMD, Watanabe S, Jans DA, Grüber G, Forwood JK, Vasudevan SG. 2019. Zika virus NS5 forms supramolecular nuclear bodies that sequester Importin-α and modulate the host immune and pro-inflammatory response in neuronal cells. ACS Infect Dis 5:932–948. doi:10.1021/acsinfecdis.8b00373 30848123

[13] Pryor MJ, Rawlinson SM, Butcher RE, Barton CL, Waterhouse TA, Vasudevan SG, Bardin PG, Wright PJ, Jans DA, Davidson AD. 2007. Nuclear localization of dengue virus nonstructural protein 5 through its importin alpha/beta-recognized nuclear localization sequences is integral to viral infection. Traffic 8:795–807. doi:10.1111/j.1600-0854.2007.00579.x 17537211

[14] De Maio FA, Risso G, Iglesias NG, Shah P, Pozzi B, Gebhard LG, Mammi P, Mancini E, Yanovsky MJ, Andino R, Krogan N, Srebrow A, Gamarnik AV. 2016. The dengue virus NS5 protein intrudes in the cellular spliceosome and modulates splicing. Pathog 12:e1005841. doi:10.1371/journal.ppat.1005841 PMC5004807 27575636

[15] Kovanich D, Saisawang C, Sittipaisankul P, Ramphan S, Kalpongnukul N, Somparn P, Pisitkun T, Smith DR. 2019. Analysis of the Zika and Japanese Encephalitis virus NS5 interactomes. J Proteome Res 18:3203–3218. doi:10.1021/acs.jproteome.9b00318 31199156

[16] Li M, Johnson JR, Truong B, Kim G, Weinbren N, Dittmar M, Shah PS, Von Dollen J, Newton BW, Jang GM, Krogan NJ, Cherry S, Ramage H. 2019. Identification of antiviral roles for the exon–junction complex and nonsense-mediated decay in flaviviral infection. Nat Microbiol 4:985–995. doi:10.1038/s41564-019-0375-z PMC6533143 30833725

[17] Shah PS, Link N, Jang GM, Sharp PP, Zhu T, Swaney DL, Johnson JR, Von Dollen J, Ramage HR, Satkamp L, et al.. 2018. Comparative flavivirus-host protein interaction mapping reveals mechanisms of Dengue and Zika virus pathogenesis. Cell 175:1931–1945. doi:10.1016/j.cell.2018.11.028 PMC6474419 30550790

[18] Li P, Wu J, Liu S, Lu R, Jiang H, Wang N, Luo M, Guo L, Xiao J, Bu L, Liu L, Xing F, Peng H, Li C, Ma L, Zhao B, Zhou Z, Guo D. 2022. The RNA polymerase of cytoplasmically replicating Zika virus binds with chromatin DNA in nuclei and regulates host gene transcription. Proc Natl Acad Sci USA 119:e2205013119. doi:10.1073/pnas.2205013119 PMC9894162 36442102

[19] Pranclova V, Nedvedova L, Kotounova E, Hönig V, Dvorakova M, Davidkova M, Bily T, Vancova M, Ruzek D, Palus M. 2024. Unraveling the role of human microglia in tick-borne encephalitis virus infection: insights into neuroinflammation and viral pathogenesis. Microbes Infect 26:105383. doi:10.1016/j.micinf.2024.105383 38942136

[20] Yau W-L, Nguyen-Dinh V, Larsson E, Lindqvist R, Överby AK, Lundmark R. 2019. Model system for the formation of tick-borne Encephalitis virus replication compartments without viral RNA replication. J Virol 93:10. doi:10.1128/JVI.00292-19 PMC6714791 31243132

[21] Selinger M, Novotný R, Sýs J, Roby JA, Tykalová H, Ranjani GS, Vancová M, Jaklová K, Kaufman F, Bloom ME, Zdráhal Z, Grubhoffer L, Forwood JK, Hrabal R, Rumlová M, Štěrba J. 2022. Tick-borne encephalitis virus capsid protein induces translational shutoff as revealed by its structural-biological analysis. J Biol Chem 298:102585. doi:10.1016/j.jbc.2022.102585 PMC9664413 36223838

[22] Abkallo HM, Kawano H, Watanabe K, Kobayashi N. 2011. A new cell-based reporter system for sensitive screening of nuclear export inhibitors. Drug Discov Ther 5:286–292. doi:10.5582/ddt.2011.v5.6.286 22466439

[23] Dosch T, Horn F, Schneider G, Krätzer F, Dobner T, Hauber J, Stauber RH. 2001. The adenovirus type 5 E1B-55K oncoprotein actively shuttles in virus-infected cells, whereas transport of E4orf6 is mediated by a CRM1-independent mechanism. J Virol 75:5677–5683. doi:10.1128/JVI.75.12.5677-5683.2001 PMC114281 11356976

[24] Gehrke R, Ecker M, Aberle SW, Allison SL, Heinz FX, Mandl CW. 2003. Incorporation of tick-borne encephalitis virus replicons into virus-like particles by a packaging cell line. J Virol 77:8924–8933. doi:10.1128/jvi.77.16.8924-8933.2003 PMC167216 12885909

[25] Teo G, Liu G, Zhang J, Nesvizhskii AI, Gingras A-C, Choi H. 2014. SAINTexpress: improvements and additional features in significance analysis of INTeractome software. J Proteomics 100:37–43. doi:10.1016/j.jprot.2013.10.023 PMC4102138 24513533

[26] Sui L, Guo X, Wang W, Xu Y, Zhao Y, Liu Q. 2024. Multi-proteomics and interactome dataset of tick-borne encephalitis virus infected host cells. Sci Data 11:1280. doi:10.1038/s41597-024-04036-y PMC11589117 39587125

[27] Gubitz AK, Mourelatos Z, Abel L, Rappsilber J, Mann M, Dreyfuss G. 2002. Gemin5, a novel WD repeat protein component of the SMN complex that binds Sm proteins. J Biol Chem 277:5631–5636. doi:10.1074/jbc.M109448200 11714716

[28] Werme K, Wigerius M, Johansson M. 2008. Tick-borne encephalitis virus NS5 associates with membrane protein scribble and impairs interferon-stimulated JAK-STAT signalling. Cell Microbiol 10:696–712. doi:10.1111/j.1462-5822.2007.01076.x 18042258

[29] Le Breton M, Meyniel-Schicklin L, Deloire A, Coutard B, Canard B, de Lamballerie X, Andre P, Rabourdin-Combe C, Lotteau V, Davoust N. 2011. Flavivirus NS3 and NS5 proteins interaction network: a high-throughput yeast two-hybrid screen. BMC Microbiol 11:234. doi:10.1186/1471-2180-11-234 PMC3215679 22014111

[30] Loughlin FE, Mansfield RE, Vaz PM, McGrath AP, Setiyaputra S, Gamsjaeger R, Chen ES, Morris BJ, Guss JM, Mackay JP. 2009. The zinc fingers of the SR-like protein ZRANB2 are single-stranded RNA-binding domains that recognize 5′ splice site-like sequences. Proc Natl Acad Sci USA 106:5581–5586. doi:10.1073/pnas.0802466106 PMC2667063 19304800

[31] Yoh SM, Cho H, Pickle L, Evans RM, Jones KA. 2007. The Spt6 SH2 domain binds Ser2-P RNAPII to direct Iws1-dependent mRNA splicing and export. Genes Dev 21:160–174. doi:10.1101/gad.1503107 PMC1770899 17234882

[32] Chen K, Liu J, Liu S, Xia M, Zhang X, Han D, Jiang Y, Wang C, Cao X. 2017. Methyltransferase SETD2-mediated methylation of STAT1 is critical for interferon antiviral activity. Cell 170:492–506. doi:10.1016/j.cell.2017.06.042 28753426

[33] Musawi S, Donnio L-M, Zhao Z, Magnani C, Rassinoux P, Binda O, Huang J, Jacquier A, Coudert L, Lomonte P, Martinat C, Schaeffer L, Mottet D, Côté J, Mari P-O, Giglia-Mari G. 2023. Nucleolar reorganization after cellular stress is orchestrated by SMN shuttling between nuclear compartments. Nat Commun 14:7384. doi:10.1038/s41467-023-42390-4 PMC10652021 37968267

[34] Thüring E-M, Hartmann C, Maddumage JC, Javorsky A, Michels BE, Gerke V, Banks L, Humbert PO, Kvansakul M, Ebnet K. 2023. Membrane recruitment of the polarity protein Scribble by the cell adhesion receptor TMIGD1. Commun Biol 6:702. doi:10.1038/s42003-023-05088-3 PMC10333293 37430142

[35] Seervai RNH, Jangid RK, Karki M, Tripathi DN, Jung SY, Kearns SE, Verhey KJ, Cianfrocco MA, Millis BA, Tyska MJ, Mason FM, Rathmell WK, Park IY, Dere R, Walker CL. 2020. The Huntingtin-interacting protein SETD2/HYPB is an actin lysine methyltransferase. Sci Adv 6:eabb7854. doi:10.1126/sciadv.abb7854 PMC7852384 33008892

[36] Yu D, Chojnowski G, Rosenthal M, Kosinski J. 2023. AlphaPulldown—a python package for protein–protein interaction screens using AlphaFold-Multimer. Bioinformatics 39:btac749. doi:10.1093/bioinformatics/btac749 PMC9805587 36413069

[37] Abramson J, Adler J, Dunger J, Evans R, Green T, Pritzel A, Ronneberger O, Willmore L, Ballard AJ, Bambrick J, et al.. 2024. Accurate structure prediction of biomolecular interactions with AlphaFold 3. Nature 630:493–500. doi:10.1038/s41586-024-07487-w PMC11168924 38718835

[38] Nielsen J, Kristensen MA, Willemoës M, Nielsen FC, Christiansen J. 2004. Sequential dimerization of human zipcode-binding protein IMP1 on RNA: a cooperative mechanism providing RNP stability. Nucleic Acids Res 32:4368–4376. doi:10.1093/nar/gkh754 PMC514376 15314207

[39] Mazeaud C, Pfister S, Owen JE, Pereira HS, Charbonneau F, Robinson ZE, Anton A, Bemis CL, Sow AA, Patel TR, Neufeldt CJ, Scaturro P, Chatel-Chaix L. 2024. Zika virus remodels and hijacks IGF2BP2 ribonucleoprotein complex to promote viral replication organelle biogenesis. eLife 13:RP94347. doi:10.7554/eLife.94347 PMC11578589 39565347

[40] Fernandez-Garcia MD, Meertens L, Chazal M, Hafirassou ML, Dejarnac O, Zamborlini A, Despres P, Sauvonnet N, Arenzana-Seisdedos F, Jouvenet N, Amara A. 2016. Vaccine and wild-type strains of Yellow fever virus engage distinct entry mechanisms and differentially stimulate antiviral immune responses. mBio 7:e01956-15. doi:10.1128/mBio.01956-15 PMC4752603 26861019

[41] Yang YHJ, Markus MA, Mangs AH, Raitskin O, Sperling R, Morris BJ. 2013. ZRANB2 localizes to supraspliceosomes and influences the alternative splicing of multiple genes in the transcriptome. Mol Biol Rep 40:5381–5395. doi:10.1007/s11033-013-2637-9 23666063

[42] Tanaka I, Chakraborty A, Saulnier O, Benoit-Pilven C, Vacher S, Labiod D, Lam EWF, Bièche I, Delattre O, Pouzoulet F, Auboeuf D, Vagner S, Dutertre M. 2020. ZRANB2 and SYF2-mediated splicing programs converging on ECT2 are involved in breast cancer cell resistance to doxorubicin. Nucleic Acids Res 48:2676–2693. doi:10.1093/nar/gkz1213 PMC7049692 31943118

[43] Ayers KL, Eggers S, Rollo BN, Smith KR, Davidson NM, Siddall NA, Zhao L, Bowles J, Weiss K, Zanni G, et al.. 2023. Variants in SART3 cause a spliceosomopathy characterised by failure of testis development and neuronal defects. Nat Commun 14:3403. doi:10.1038/s41467-023-39040-0 PMC10256788 37296101

[44] Lopez-Denman AJ, Mackenzie JM. 2017. The importance of the nucleus during Flavivirus replication. Viruses 9:14. doi:10.3390/v9010014 PMC5294983 28106839

[45] Tay MYF, Smith K, Ng IHW, Chan KWK, Zhao Y, Ooi EE, Lescar J, Luo D, Jans DA, Forwood JK, Vasudevan SG. 2016. The C-terminal 18 amino acid region of Dengue virus NS5 regulates its subcellular localization and contains a conserved arginine residue essential for infectious virus production. Pathog 12:e1005886. doi:10.1371/journal.ppat.1005886 PMC5021334 27622521

[46] Rawlinson SM, Pryor MJ, Wright PJ, Jans DA. 2009. CRM1-mediated nuclear export of dengue virus RNA polymerase NS5 modulates interleukin-8 induction and virus production. J Biol Chem 284:15589–15597. doi:10.1074/jbc.M808271200 PMC2708855 19297323

[47] Lopez-Denman AJ, Russo A, Wagstaff KM, White PA, Jans DA, Mackenzie JM. 2018. Nucleocytoplasmic shuttling of the West Nile virus RNA-dependent RNA polymerase NS5 is critical to infection. Cell Microbiol 20:e12848. doi:10.1111/cmi.12848 29582535

[48] Fraser JE, Watanabe S, Wang C, Chan WKK, Maher B, Lopez-Denman A, Hick C, Wagstaff KM, Mackenzie JM, Sexton PM, Vasudevan SG, Jans DA. 2014. A nuclear transport inhibitor that modulates the unfolded protein response and provides *in vivo* protection against lethal dengue virus infection. J Infect Dis 210:1780–1791. doi:10.1093/infdis/jiu319 24903662

[49] Wagstaff KM, Sivakumaran H, Heaton SM, Harrich D, Jans DA. 2012. Ivermectin is a specific inhibitor of importin α/β-mediated nuclear import able to inhibit replication of HIV-1 and dengue virus. Biochem J 443:851–856. doi:10.1042/BJ20120150 PMC3327999 22417684

[50] Wang C, Yang SNY, Smith K, Forwood JK, Jans DA. 2017. Nuclear import inhibitor N -(4-hydroxyphenyl) retinamide targets Zika virus (ZIKV) nonstructural protein 5 to inhibit ZIKV infection. Biochem Biophys Res Commun 493:1555–1559. doi:10.1016/j.bbrc.2017.10.016 28988109

[51] Petit MJ, Kenaston MW, Pham OH, Nagainis AA, Fishburn AT, Shah PS. 2021. Nuclear dengue virus NS5 antagonizes expression of PAF1-dependent immune response genes. Pathog 17:e1010100. doi:10.1371/journal.ppat.1010100 PMC8641875 34797876

[52] Miao M, Yu F, Wang D, Tong Y, Yang L, Xu J, Qiu Y, Zhou X, Zhao X. 2019. Proteomics profiling of host cell response via protein expression and phosphorylation upon dengue virus infection. Virol Sin 34:549–562. doi:10.1007/s12250-019-00131-2 PMC6814695 31134586

[53] Walter Z, Li M, Molho M, Berish L, Isopi A, O’Mara M, Dittmar M, Nwaezeapu C, Richards A, McCullagh M, Krogan NJ, Cherry S, Johnson JR, Ramage H. 2025. An integrated proteomics approach identifies phosphorylation sites on viral and host proteins that regulate West Nile virus infection. Cell Rep 44:115728. doi:10.1016/j.celrep.2025.115728 PMC12180539 40381193

[54] Chazal M, Beauclair G, Gracias S, Najburg V, Simon-Lorière E, Tangy F, Komarova AV, Jouvenet N. 2018. RIG-I recognizes the 5’ region of Dengue and Zika virus genomes. Cell Rep 24:320–328. doi:10.1016/j.celrep.2018.06.047 29996094

[55] Gagnon KT, Li L, Janowski BA, Corey DR. 2014. Analysis of nuclear RNA interference in human cells by subcellular fractionation and Argonaute loading. Nat Protoc 9:2045–2060. doi:10.1038/nprot.2014.135 PMC4251768 25079428

[56] Bruderer R, Bernhardt OM, Gandhi T, Xuan Y, Sondermann J, Schmidt M, Gomez-Varela D, Reiter L. 2017. Optimization of experimental parameters in data-independent mass spectrometry significantly increases depth and reproducibility of results. Mol Cell Proteomics 16:2296–2309. doi:10.1074/mcp.RA117.000314 PMC5724188 29070702

[57] Choi M, Chang C-Y, Clough T, Broudy D, Killeen T, MacLean B, Vitek O. 2014. MSstats: an R package for statistical analysis of quantitative mass spectrometry-based proteomic experiments. Bioinformatics 30:2524–2526. doi:10.1093/bioinformatics/btu305 24794931

[58] Teo G, Koh H, Fermin D, Lambert J-P, Knight JDR, Gingras A-C, Choi H. 2016. SAINTq: scoring protein-protein interactions in affinity purification - mass spectrometry experiments with fragment or peptide intensity data. Proteomics 16:2238–2245. doi:10.1002/pmic.201500499 27119218

[59] Perez-Riverol Y, Bandla C, Kundu DJ, Kamatchinathan S, Bai J, Hewapathirana S, John NS, Prakash A, Walzer M, Wang S, Vizcaíno JA. 2025. The PRIDE database at 20 years: 2025 update. Nucleic Acids Res 53:D543–D553. doi:10.1093/nar/gkae1011 PMC11701690 39494541

