# Supplementary material for: Nuclear activities and interactome of the NS5 protein of tick-borne encephalitis virus
Source: J Virol. 2026 Aug 10;100(9):e00810-26. doi: 10.1128/jvi.00810-26 (PMC13595970; doi:10.1128/jvi.00810-26)
Supplement: Supplementary file 1 [file jvi.00810-26-s0001.pdf]

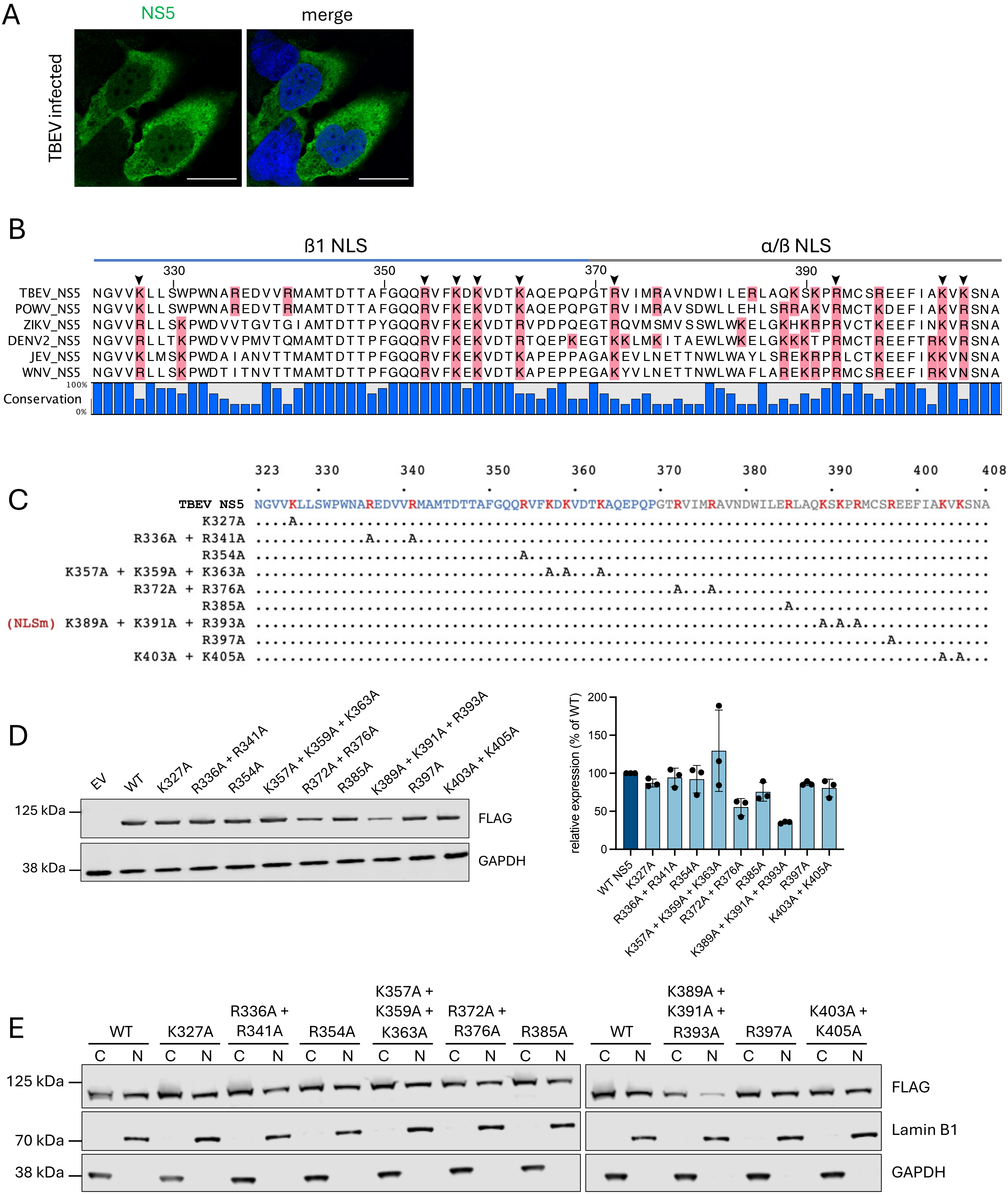

A
NS5
merge
TBEV infected
B
β1 NLS
α/β NLS
TBEV_NS5
POWV_NS5
ZIKV_NS5
DENV2_NS5
JEV_NS5
WNV_NS5
Conservation
C
TBEV NS5 NGVVKLLSWPWNAREDVVRMAMTDTTAFGQQRVFKDKVDTKAQEPQPGTRVIMRAVNDWILERLAQKSKPRMCSREEFIAKVKSNA
K327A
R336A + R341A
R354A
K357A + K359A + K363A
R372A + R376A
R385A
(NLSm) K389A + K391A + R393A
R397A
K403A + K405A
D
EV
WT
125 kDa
38 kDa
FLAG
GAPDH
relative expression (% of WT)
WT NS5
E
C
N
Lamin B1
70 kDa

Figure S1

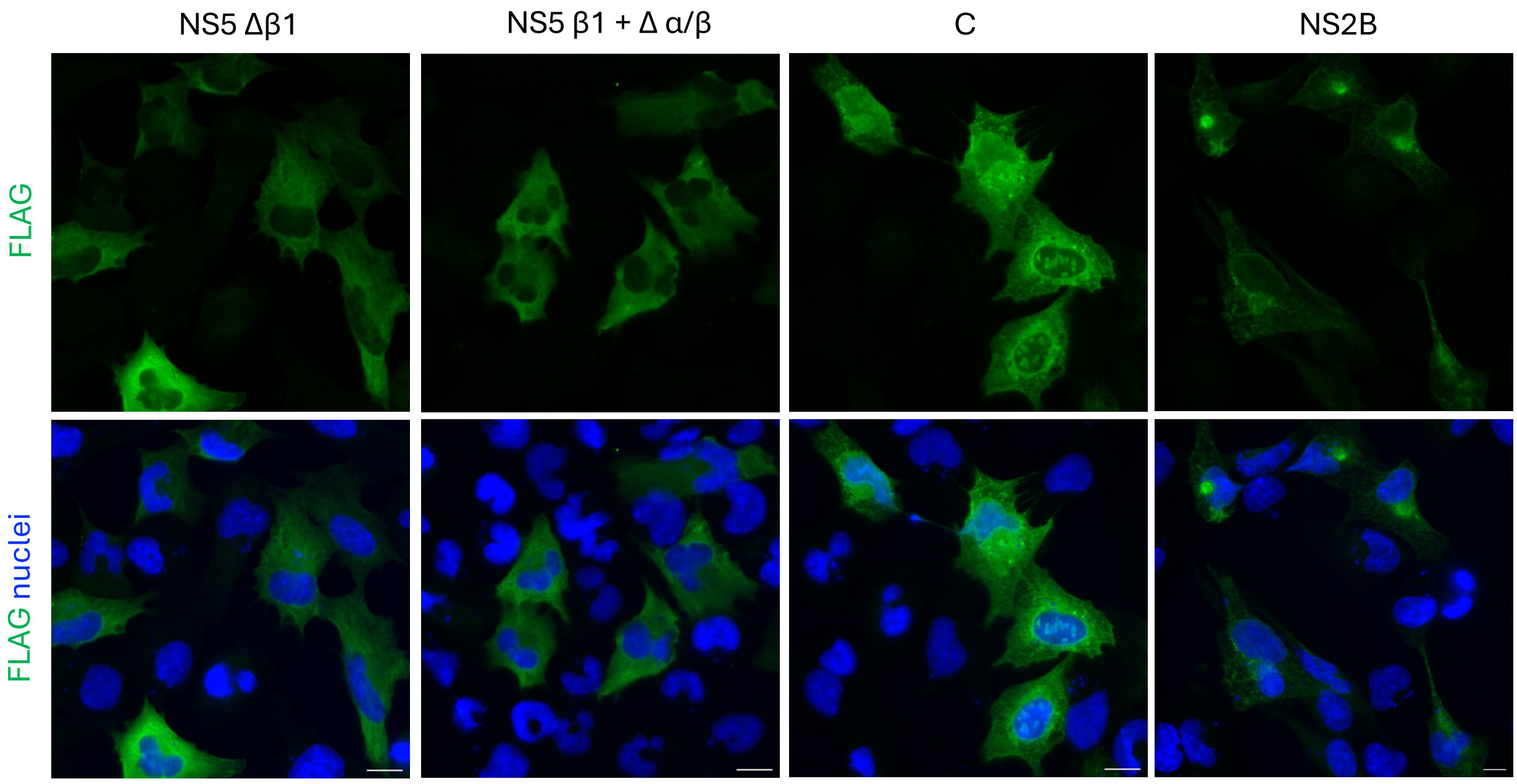

Figure S2

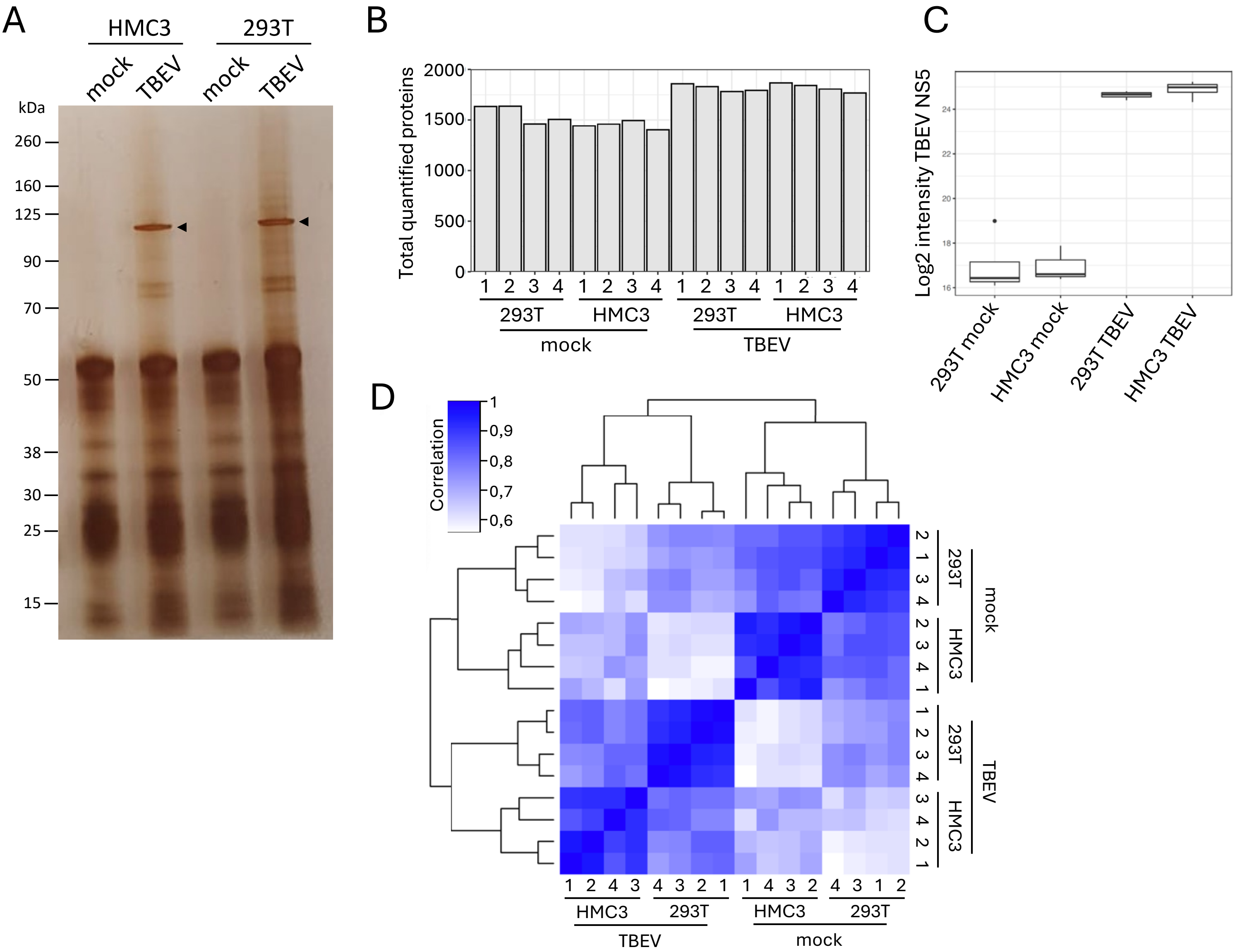
A
HMC3
293T
mock
TBEV
mock
TBEV
kDa
260
160
125
90
70
50
38
30
25
15
B
Total quantified proteins
2000
1500
1000
500
0
1 2 3 4 1 2 3 4 1 2 3 4 1 2 3 4
293T
HMC3
293T
HMC3
mock
TBEV
C
Log2 intensity TBEV NS5
293T mock
HMC3 mock
293T TBEV
HMC3 TBEV
D
Correlation
1
0,9
0,8
0,7
0,6
1 2 4 3 4 3 2 1 1 4 3 2 4 3 1 2
HMC3
293T
HMC3
293T
TBEV
mock
2 1 3 4
293T
2 3 4 1
HMC3
mock
1 2 3 4
293T
3 4 2 1
HMC3
TBEV

Figure S3

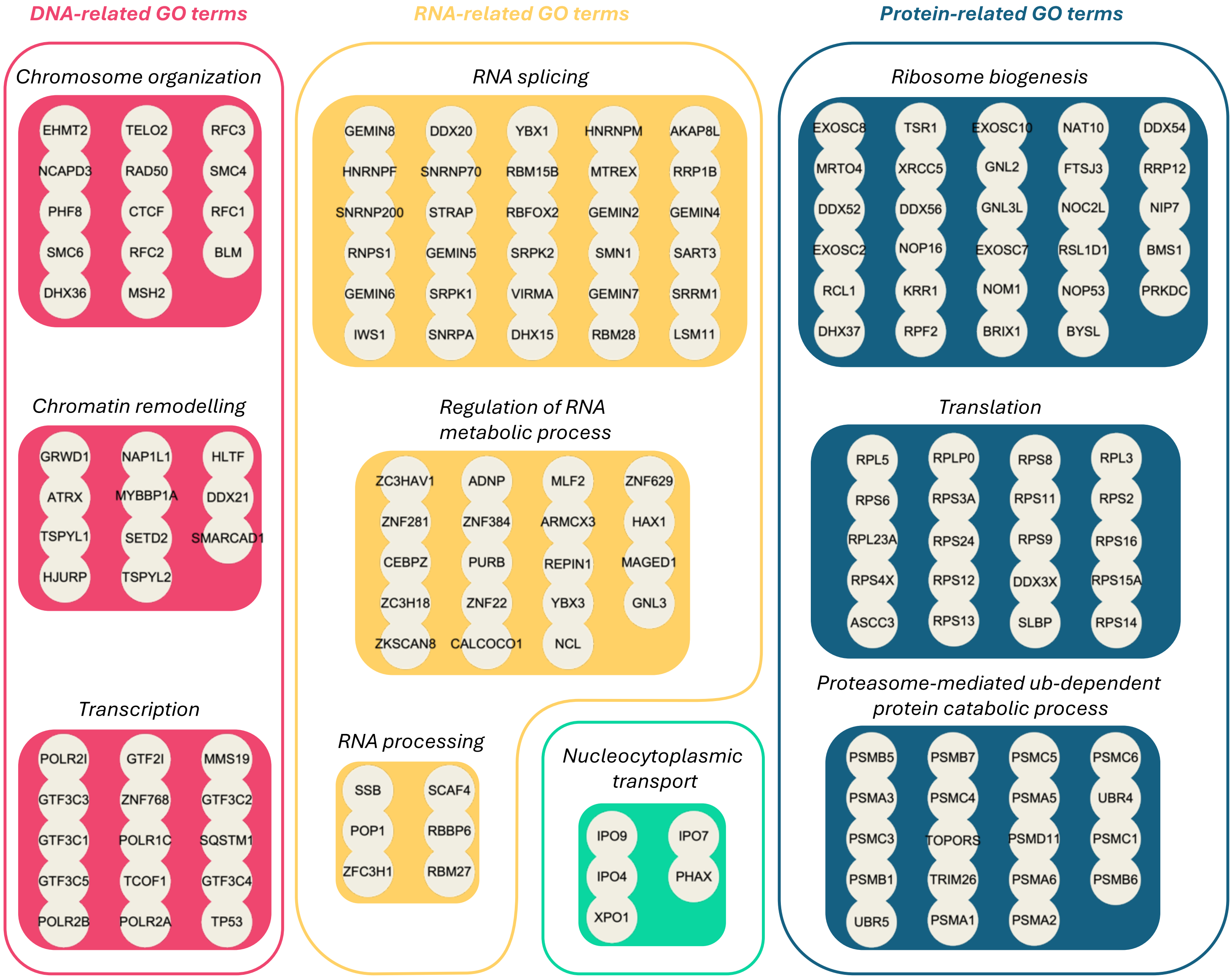

DNA-related GO terms
RNA-related GO terms
Protein-related GO terms
Chromosome organization
EHMT2 TELO2 RFC3 NCAPD3 RAD50 SMC4 PHF8 CTCF RFC1 SMC6 RFC2 BLM DHX36 MSH2
Chromatin remodelling
GRWD1 NAP1L1 HLTF ATRX MYBBP1A DDX21 TSPYL1 SETD2 SMARCAD1 HJURP TSPYL2
Transcription
POLR2I GTF2I MMS19 GTF3C3 ZNF768 GTF3C2 GTF3C1 POLR1C SQSTM1 GTF3C5 TCOF1 GTF3C4 POLR2B POLR2A TP53
RNA splicing
GEMIN8 DDX20 YBX1 HNRNPM AKAP8L HNRNPF SNRNP70 RBM15B MTREX RRP1B SNRNP200 STRAP RBFOX2 GEMIN2 GEMIN4 RNPS1 GEMIN5 SRPK2 SMN1 SART3 GEMIN6 SRPK1 VIRMA GEMIN7 SRRM1 IWS1 SNRPA DHX15 RBM28 LSM11
Regulation of RNA metabolic process
ZC3HAV1 ADNP MLF2 ZNF629 ZNF281 ZNF384 ARMCX3 HAX1 CEBPZ PURB REPIN1 MAGED1 ZC3H18 ZNF22 YBX3 GNL3 ZKSCAN8 CALCOCO1 NCL
RNA processing
SSB SCAF4 POP1 RBBP6 ZFC3H1 RBM27
Nucleocytoplasmic transport
IPO9 IPO7 IPO4 PHAX XPO1
Ribosome biogenesis
EXOSC8 TSR1 EXOSC10 NAT10 DDX54 MRTO4 XRCC5 GNL2 FTSJ3 RRP12 DDX52 DDX56 GNL3L NOC2L NIP7 EXOSC2 NOP16 EXOSC7 RSL1D1 BMS1 RCL1 KRR1 NOM1 NOP53 PRKDC DHX37 RPF2 BRIX1 BYSL
Translation
RPL5 RPLP0 RPS8 RPL3 RPS6 RPS3A RPS11 RPS2 RPL23A RPS24 RPS9 RPS16 RPS4X RPS12 DDX3X RPS15A ASCC3 RPS13 SLBP RPS14
Proteasome-mediated ub-dependent protein catabolic process
PSMB5 PSMB7 PSMC5 PSMC6 PSMA3 PSMC4 PSMA5 UBR4 PSMC3 TOPORS PSMD11 PSMC1 PSMB1 TRIM26 PSMA6 PSMB6 UBR5 PSMA1 PSMA2

Figure S4

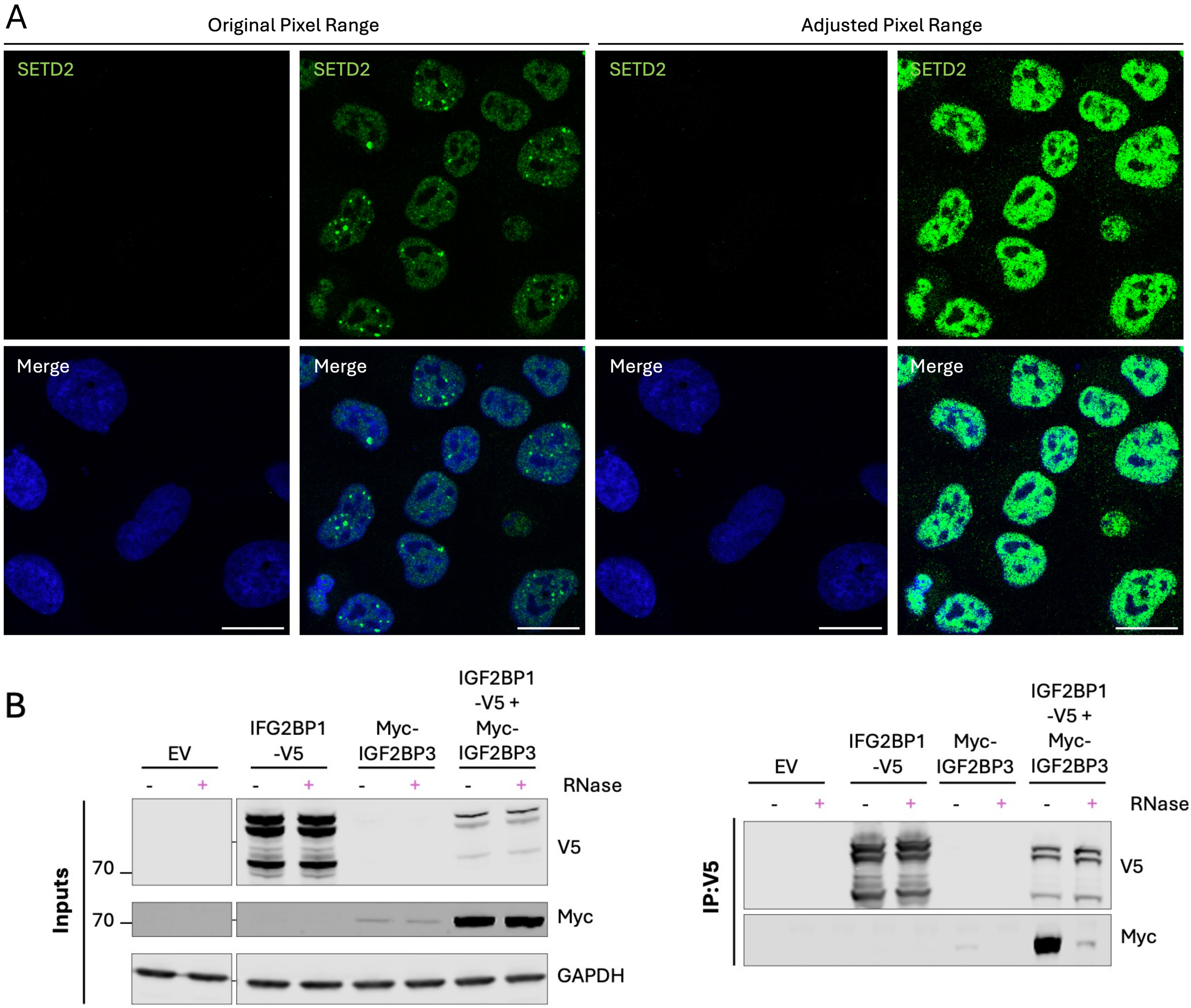
A
Original Pixel Range
Adjusted Pixel Range
SETD2
Merge
B
EV
IFG2BP1 -V5
Myc- IGF2BP3
IGF2BP1 -V5 + Myc- IGF2BP3
RNase
Inputs
70
V5
Myc
GAPDH
IP:V5

Figure S5
