# Supplementary material for: Nuclear activities and interactome of the NS5 protein of tick-borne encephalitis virus
Source: J Virol. 2026 Aug 10;100(9):e00810-26. doi: 10.1128/jvi.00810-26 (PMC13595970; doi:10.1128/jvi.00810-26)
Supplement: Supplementary file 2 [file jvi.00810-26-s0002.docx]

**Supplementary figure legends**

**Figure S1. Nuclear localization of TBEV NS5.**

A. Huh7 cells were infected with TBEV at an MOI of 1. After 24 hours, subcellular localization of NS5 was determined by immunofluorescence microscopy using antibodies against TBEV NS5 (green). Nuclei were stained using DAPI (blue) and images were acquired using a confocal microscope. Scale bars 20 µm.

B. Sequence alignment of NLS importin β1- and α/β-interacting domains of several orthoflavivirus NS5 proteins. Positively charged residues are highlighted in red and arrows indicate conserved ones.

C. Representation of TBEV NS5 point mutants generated by mutagenesis. Positively charged residues (in red) were mutated to alanine.

D. 293T cells were transfected with plasmids encoding TBEV FLAG-NS5 WT or mutants. After 24 hours, whole-cell lysates were analyzed by western blot using antibodies against FLAG and GAPDH. Expression levels of NS5 proteins were measured using ImageStudio and normalized to the WT NS5 level, which was set to 100%.

E. 293T cells were transfected with plasmids encoding TBEV FLAG-NS5 WT or mutants. After 24 hours, NS5 subcellular localization was determined by analyzing cytoplasmic (C) and nuclear (N) fractions by western blot using antibodies against FLAG, GAPDH and Lamin B1.

**Figure S2. Localization of TBEV NS5 mutants, C and NS2B proteins in transfected HMC3 cells.**

HMC3 cells were transfected with plasmids expressing FLAG-tagged NS5 mutants, C or NS2B. After 24 hours, subcellular localization of these proteins was determined by immunofluorescence microscopy using an antibody recognizing FLAG (green). Nuclei were stained using DAPI (blue) and images were acquired using an epifluorescence microscope. Scale bars 10 µm.

**Figure S3. Mass spectrometry complementary data.**

A. Representative silver staining of immunoprecipitated samples from HMC3 and 293T infected cells. Silver-stained bands corresponding to NS5 are indicated by arrows.

B. Number of unique proteins quantified for each sample.

C. Box plot of TBEV NS5 protein intensities quantified for each condition.

D. Clustered heatmap of Pearson correlation comparing protein log_2_ intensity profiles for each pair of samples.

**Figure S4. Gene Ontology analysis of TBEV NS5 nuclear partners.**

Categorical representation of Gene Ontology biological processes enriched among nuclear host factors interacting with the TBEV NS5 protein in infected cells.

**Figure S5. Further characterization of the interactions between NS5 and its partners using confocal microscopy and RNAse A treatment.**

A. HMC3 cells were stained with antibodies against SETD2 (green) and DAPI (blue). Images were acquired using a confocal microscope. Acquisition settings were based on cells stained with secondary antibodies only (control). Original and adjusted pixel ranges are shown. Scale bars 10 µm.

B. 293T cells were transfected for 24 h with plasmids encoding IGF2BP1-V5 and myc-IGF2BP3, or an empty vector as control (EV). Cell lysates were treated with RNase A or left untreated and were immunoprecipitated using V5 antibodies. Western blot analyses were performed on whole cell lysates (inputs) and V5-immunoprecipitates (IP) using the indicated antibodies.

**Supplementary Tables**

Table S1. SAINTq analysis of TBEV NS5 DIA-MS data

Table S2. Global proteomics of TBEV-infected cells

Table S3. AlphaFold Pulldown TBEV NS5 and high confidence partners

Table S4. Primers for cloning

Table S5. Primers for qPCR
